# Cannabinoid efficacy for pain: dose, chronicity and route

**DOI:** 10.3389/fnins.2026.1888863

**Published:** 2026-08-13

**Authors:** Kaylin J. Ellioff, Madalyn Critz, Nephi Stella, Michael R. Bruchas, Benjamin B. Land

**Affiliations:** 1 Department of Pharmacology, University of Washington, Seattle, WA, United States; 2 Center for the Neurobiology of Addiction, Pain, and Emotion, University of Washington, Seattle, WA, United States; 3 Department of Psychiatry and Behavioral Sciences, University of Washington, Seattle, WA, United States; 4 Department of Anesthesiology and Pain Medicine, University of Washington, Seattle, WA, United States; 5 Department of Bioengineering, University of Washington, Seattle, WA, United States

**Keywords:** behavior, cannabidiol, Delta-9-tetrahydrocannabinol, entourage, machine-learning, pain, terpene

## Abstract

*Cannabis*-based therapies are widely used for chronic pain, yet their mechanisms and therapeutic windows remain incompletely defined. This review synthesizes preclinical and clinical evidence on how dose, route of administration, treatment duration, and chemical composition shape analgesic efficacy and adverse-effect liability for Δ^9^-tetrahydrocannabinol (THC), cannabidiol (CBD), and select *cannabis*-derived terpenes. Across rodent pain models, acute THC reliably produces antinociception, but its therapeutic window is narrow because analgesic doses overlap with CB_1_ receptor–mediated side effects such as sedation, hypothermia, hyperphagia, and motor impairment. Repeated THC exposure leads to tolerance and dependence. In contrast, CBD shows limited acute efficacy in naïve and inflammatory models but demonstrates more consistent benefit with repeated dosing in neuropathic and chemotherapy-induced pain, often without cannabimimetic adverse effects. Terpenes such as linalool, β-caryophyllene, myrcene, limonene, α-terpineol, and α-bisabolol exhibit independent antinociceptive and anti-inflammatory properties and are thought to pharmacologically interact with cannabinoids in a dose-, ratio-, and route-dependent “entourage” effect that either enhance or constrain therapeutic benefit. This review also focuses on integrating machine learning–based behavioral phenotyping of rodents to refine cannabinoid analgesia preclinical research. Computer vision pose-estimation and unsupervised clustering approaches enable high-resolution quantification of spontaneous and evoked natural behaviors, allowing the analytical dissociation of true analgesia from sedation, ataxia, or reduced exploration. By coupling these behavioral pipelines with pharmacokinetic and circuit-level analyses, emerging frameworks will define therapeutic windows with greater precision and improve the translational relevance of cannabinoid-based pain therapeutics.

## 1 Introduction

Cannabinoid-based therapeutics are among the most widely used treatments for pain indications, with nearly one-third of adults with chronic pain using *Cannabis* for pain management (Bicket et al., 2023). *Cannabis* remains one of the least mechanistically resolved interventions, with variable outcomes across medical indications, patient populations, products, and dosing regimens. Phytocannabinoids exhibit pharmacological promiscuity that extends beyond cannabinoid 1 receptor (CB_1_R), cannabinoid 2 receptor (CB_2_R), transient receptor potential ankyrin and vanilloid (TRPA, TRPV) channels, and G protein-coupled receptor 55 (GPR55), engaging additional targets including cyclooxygenase-2 (COX2), the orphan receptors GPR3, GPR6, GPR12, GPR18, and GPR119, peroxisome proliferator-activated receptors (PPARs), and 5-HT1A serotonin receptors (Grotenhermen, 2004; Starowicz and Finn, 2017; Narouze, 2021; Mlost et al., 2020; Murillo-Rodriguez et al., 2017; Miranda-Cortés et al., 2023; Singh et al., 2024; Lecamp et al., 2026). A comprehensive treatment of this polypharmacology is beyond the scope of the present review; this review therefore focuses on dose-, route-, chronicity-, and composition-dependent determinants of analgesic efficacy. Given the breadth of the phytocannabinoid and synthetic cannabinoid landscape, we focus most extensively on THC, CBD, and *Cannabis*-derived terpenes to allow an in-depth look at variables that cut across all three classes of compound (e.g., dose, route, and chronicity). An expansive, systematic treatment of minor phytocannabinoids (e.g., cannabigerol, cannabinol, cannabichromene) and/or synthetic analogs (e.g., dronabinol, nabilone) is a necessary complement that is outside the scope of the present narrative.

**FIGURE 1:**
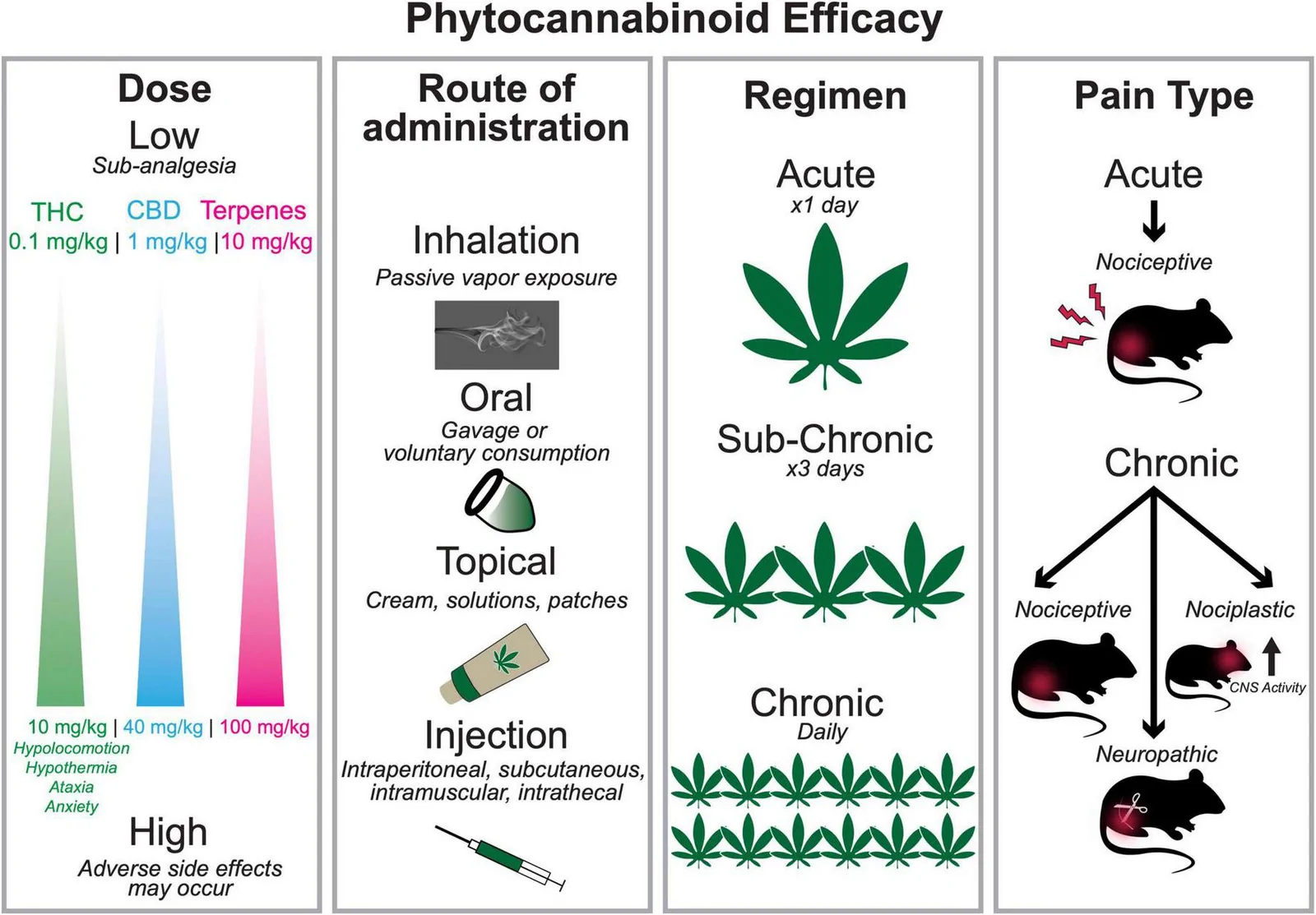
Phytocannabinoid efficacy. Four main variables shape the analgesic outcome of THC, CBD, and terpene administration in rodent pain research. Dose (left): each compound has a distinct effective dose range: approximately 0.1–10 mg/kg for THC, 1–40 mg/kg for CBD, and 10–100 mg/kg for terpenes, below which analgesia is not reliably achieved (sub-analgesic) and above which cannabimimetic adverse effects including hypolocomotion, hypothermia, ataxia, and anxiety may emerge. Route of administration (second from left): inhalation (passive vapor exposure), oral (gavage or voluntary consumption), topical (creams, solutions, or patches), and injection (intraperitoneal, subcutaneous, intramuscular, or intrathecal). Regimen (second from right): dosing chronicity: acute (single day), sub-chronic (approximately 3 days), or chronic (daily). Pain type (right): acute nociceptive pain and chronic pain states (nociceptive, neuropathic, and nociplastic) differentially engage peripheral and central sensitization mechanisms, including CNS activity in nociplastic conditions, and each respond to cannabinoids with distinct dose- and time-course profiles. Together, these four variables interact to determine whether a given cannabinoid or terpene produces meaningful analgesia in a preclinical model, and mismatches across these dimensions are a primary driver of inconsistency in the preclinical literature.

Since state-level legalization of *Cannabis* in the United States has expanded access to *Cannabis*-based products beginning in 2012, preclinical work has rapidly grown and established that phytocannabinoids such as Δ^9^-tetrahydrocannabinol (THC), cannabidiol (CBD), and cannabis-derived terpenes can modulate nociceptive, inflammatory, and affective components of pain. However, even in preclinical work, analgesic efficacy varies widely as a function of dose, route of administration, treatment duration, pain type and chemical composition (Figure 1). As detailed in the sections below, these dose- and route-dependent effects differ across compound classes. For example: THC’s therapeutic window narrows at doses that also produce psychomotor impairment (English et al., 2024), CBD’s efficacy depends more on treatment chronicity than acute dose (Silva-Cardoso et al., 2021), and terpene-mediated analgesia can vary by delivery route and formulation, in some cases even reversing direction (Kaimoto et al., 2016). We return to each of these compound-specific relationships in the THC, CBD, and terpenes sections that follow. These variables shape not only the magnitude and duration of analgesia but also side-effect profiles, including classic CB_1_R-mediated outcomes like hypothermia, catalepsy, and sedation at higher systemic THC doses. CB_1_R is highly expressed in pain-processing regions like the dorsal horn of the spinal cord, dorsal root ganglia, periaqueductal gray, and anterior cingulate cortex, where its G_*i*/*o*_-coupled signaling suppresses presynaptic neurotransmitter release and reduces neuronal excitability (Starowicz and Finn, 2017). CBD does not bind to CB_1_R with high affinity (it does act as a negative allosteric modulator), but instead is thought to modulate pain via transient receptor potential vanilloid 1 (TRPV1) desensitization, GPR55 antagonism, and inhibition of endocannabinoid reuptake, with expression of its primary targets across sensory neurons and spinal interneurons (Mlost et al., 2020; Starowicz and Finn, 2017). Terpenes such as linalool, β-caryophyllene, and myrcene engage overlapping targets including transient receptor potential ankyrin 1 (TRPA1), CB_2_R and opioid receptors, with expression mainly in peripheral sensory and immune cells (Liktor-Busa et al., 2021). among cannabinoids and terpenes, coined the “entourage effect”, add another layer of complexity, since some combinations enhance analgesia or widen the therapeutic window, whereas others are either sub-additive or shift side-effect liability. It should be noted that the entourage effect remains scientifically contested. Preclinical evidence supports pharmacodynamic interactions between *Cannabis* constituents; however, robust mechanistic demonstration in humans is limited. Terpenes such as linalool, β-caryophyllene, myrcene, limonene, α-terpineol, and α-bisabolol can thus shape cannabinoid pharmacodynamics through receptor-level and pharmacokinetic interactions, producing ‘entourage’ effects that are dose-, ratio-, and route-dependent. Because of the diversity of cannabis strains and novel formulations, it is increasingly important to understand how these parameters determine therapeutic window, side-effects and translational relevance.

A challenge for both the pain and cannabinoid fields of research is that the doses required for analgesia often overlap with those that produce sedation, motor impairment, and psychoactive effects, making it difficult to define a true therapeutic window. This is compounded by dose-response relationships that are frequently narrow and non-monotonic or cooperative, meaning that small changes in dose can shift a compound from analgesic to impairing, or produce no additional benefit despite increasing exposure. THC demonstrates this acutely: it produces antinociception when dosed acutely at low-to-moderate doses, but tolerance, psychomotor impairment, and cannabimimetic side effects emerge quickly with repeated or escalating exposure. On the other hand, in many cases CBD induces little acute analgesia, but has consistent efficacy with subchronic or chronic administration. Clarifying CBD’s dose-efficacy relationships, and how they differ from THC’s, remains a major goal in our field of research. Entourage interactions such as THC:CBD, CBD:terpene, THC:terpene, and combinations involving minor cannabinoids introduce additional complexity, but also a polypharmacology therapeutic opportunity. Evidence demonstrates specific pairings can enhance antinociception, reduce CB_1_R-mediated side effects, or improve affective outcomes, even though mechanistic receptor-level evidence is still heterogeneous. Advances in behavioral phenotyping and neuroimaging are starting to resolve some of this variability. Classical assays such as von Frey, hot plate, Hargreaves, and tail flick assays provide low-resolution, evoked readouts that can be subjective or inconsistent in nature across labs. New high-throughput, automated, and unbiased approaches which leverage Computer vision machine learning based kinematic quantification and unsupervised behavioral clustering offer greater resolution to nociceptive and affective states in rodents. These approaches, coupled with advances in calcium and whole-brain imaging, make it possible to link specific cannabinoid regimens to defined circuit-level physiological changes, providing preclinical biosignature readouts which more closely mirror human neuroimaging and behavioral endpoints.

At the same time, the mechanisms that govern cannabinoid pharmacokinetics (PK): absorption, distribution, metabolism and clearance, remain critical determinants of therapeutic range. Oral, inhaled, intrathecal, and transdermal routes each result in distinct plasma and brain concentration-time profiles and different metabolite spectra, which in turn influence onset, peak effect, duration, and the balance between analgesia and adverse events. Integrating these PK features with behavioral, imaging and molecular data is critical to establish a preclinical framework that guides clinical translation. In this review, we synthesize both preclinical and clinical evidence on how dose, regimen and route of administration play into cannabinoid-based analgesia with a focus on THC, CBD, terpenes, pharmacodynamic and PK entourage mechanisms, and emerging technologies for pain quantification. A primary goal in the field at large is to better define parameters that expand or constrain the therapeutic dose range of cannabinoid-based therapies and to highlight barriers and novel tools that will shape the next generation of cannabinoid research.

## 2 Cannabis use

### 2.1 Cannabis and cannabinoids for pain relief in preclinical studies

This section focuses on CBD, THC, cannabis-derived terpenes, and combinations of these compounds with each other and with minor cannabinoids. Although the entourage effect has been studied in mood and anxiety disorders (Ferber et al., 2020), its analgesic relevance in preclinical pain models remains less well characterized, a gap this section addresses. In 2009, Wagner and Ulrich-Merzenich proposed four mechanisms of synergy: multitarget effects, pharmacokinetic effects, agent interactions, and adverse event manipulation. In *in vitro* assays, THC acts as a partial agonist at CB_1_R (∼41% of 2-AG response), 16 tested-terpenes activate CB_1_R (10–50% of THC response), and THC + terpene mixtures enhanced CB_1_R activation beyond THC as single agent, with several combinations (i.e., with borneol, limonene, and sabinene) showing synergistic effects (Raz et al., 2023).

Dose plays a major role in how these compounds interact and the growing use of high-potency *Cannabis*-based products has shifted toward higher THC content which may be at the expense of CBD, terpenes and minor cannabinoid compounds (ElSohly et al., 2016). Within the preclinical studies we later discuss, phytocannabinoids, terpenes, and cannabinoid-modulating compounds demonstrate strong but variable analgesic efficacy with differences largely attributable to dose, route of administration, treatment duration (acute versus chronic), and the preclinical pain model used. Pharmacokinetic factors, like absorption and first-pass metabolism after oral dosing, rapid brain entry with inhalation, and restricted distribution after intrathecal or intraplantar administration, shape the onset magnitude and duration of analgesia in addition to various side-effect profiles (Huestis, 2007).

#### 2.1.1 THC

Across inflammatory, neuropathic, visceral, and chemotherapy-induced pain models, acute THC administered intraperitoneally (i.p.), subcutaneously (s.c.), orally, intrathecally, or via vapor reliably produce analgesia at roughly 0.3–10 mg/kg systemically or comparable vapor exposures (English et al., 2024; Javadi-Paydar et al., 2018; Jenkins et al., 2025; Harris et al., 2019; Craft et al., 2023; Mitchell et al., 2021; Casey et al., 2017; Pearl-Dowler et al., 2023; Henderson-Redmond et al., 2021; King et al., 2017; Deng et al., 2015; Morris et al., 2024). Higher systemic doses produce more intense central nervous system effects including the CB_1_R-mediated tetrad of hypothermia, catalepsy, reduced locomotion, and sedation, narrowing the therapeutic window (Martin et al., 1991).

In pain naïve animals, i.p. delivery of either Δ^8^- or Δ^9^-THC (1.8–5.6 mg/kg) dose-dependently increased tail-withdrawal latency (Morris et al., 2024). Maximal tail-flick analgesia was achieved at 30 mg/kg i.p. and at approximately 10.5 mg/kg via THC-contained gelatin, with both routes producing cannabimimetic side effects at analgesic doses (English et al., 2024). THC vapor inhalation (12.5–200 mg/mL) similarly reduced body temperature and increased tail-withdrawal latency in a concentration-dependent manner (Javadi-Paydar et al., 2018). Taken together, cannabimimetic side effects like hypothermia, catalepsy, hypolocomotion, and antinociception (classical tetrad), were seen at doses that produced maximal analgesia, demonstrating the challenges of separating analgesic from sedative or motor-impairing actions.

In carrageenan-induced inflammatory pain, oral THC (1, 3, and 10 mg/kg) dose-dependently reduced thermal hyperalgesia (Hargreaves) and mechanical allodynia (von Frey) with stronger effects in females and a pro-inflammatory effect of the lowest dose in males, emphasizing interactions between sex and dose-specific responses (Jenkins et al., 2025). THC at 5 and 10 mg/kg i.p. increased hot plate latency and at 2.5–10 mg/kg increased tail-flick latency and reduced abdominal writhing in acetic-acid-induced abdominal writhing (Harris et al., 2019), again demonstrating robust antinociception in acute inflammatory and visceral assays. In Complete Freund’s Adjuvant (CFA)-induced hindpaw inflammation, THC-dominant vapor (200 or 400 mg/mL) acutely reduced mechanical allodynia, improved weight-bearing and locomotion, and reduced edema (Craft et al., 2023). Although, with repeated twice-daily exposure over 3 days, only anti-allodynia persisted, indicating selective tolerance of weight-bearing, locomotion, and anti-edema (Craft et al., 2023).

Intrathecally, THC produces antihyperalgesia in a partial sciatic nerve injury (pSNL) mice and produced a dose-dependent reduction in formalin- and CFA-evoked nociception (Gadotti et al., 2023). A separate study showed that intrathecal THC dose-dependently reduced chronic constriction injury (CCI)-induced mechanical and cold allodynia with ED_50_s of 14 and 20 nmol without producing cannabinoid-like side effects (Casey et al., 2022). This highlights the potential utility of the dissociation between analgesia and central adverse events with spinal delivery. In a chronic constriction injury (CCI) neuropathic pain model, oral THC (0.3–56 mg/kg) tested 11–14 days after injury produced dose-dependent reductions in mechanical allodynia and acetone-evoked responses, with ED_50_s of 6.3 and 5.5 mg/kg, but side effects at similar potency levels, again constraining the therapeutic index (Mitchell et al., 2021). Subcutaneous THC alleviated CCI-induced mechanical and thermal allodynia with an ED_50_ of 2.7–4.3 mg/kg, but rotarod impairment with an EC_50_ of 6–15 mg/kg, suggesting a modest but real separation between analgesic and motor effects (Casey et al., 2017). Oral THC (1–10 mg/kg) in another CCI study reduced mechanical and cold allodynia (Mitchell et al., 2021), and in spared nerve injury (SNI) a single i.p. dose of 2.5–5 mg/kg decreased mechanical allodynia; in morphine-tolerant SNI rats, a single 2.5 mg/kg THC injection still reduced allodynia, indicating preserved efficacy even in an opioid-tolerance state (Pearl-Dowler et al., 2023).

THC also demonstrates efficacy in systemic and disease-specific neuropathic conditions. In humanized sickle cell disease mice, acute THC (0.1–3 mg/kg i.p.) dose-dependently reduced mechanical and cold hypersensitivity, with tail-flick antinociception observed (Mabou Tagne et al., 2024). Subchronic dosing (1 or 3 mg/kg daily for 14 days) maintained mechanical but not cold analgesia without altering anxiety-like or cognitive behaviors (Mabou Tagne et al., 2024). Chronic oral THC (1 mg/15 mL in gelatin, ∼5 g consumed per day) reduced allodynia, thermal hyperalgesia, and pain-related ultrasonic broadband clicks over 3 weeks, demonstrating durable behavioral benefit in neuropathic pain (Abraham et al., 2020). In an endometriosis model, chronic THC (2 mg/kg s.c. daily) alleviated abdominal mechanical hypersensitivity and pain unpleasantness, prevented endometriosis-associated cognitive deficits, and suppressed growth of ectopic endometrial cysts without tolerance to antinociception, whereas acute THC (1.25–5 mg/kg s.c.) dose-dependently reduced mechanical hypersensitivity (Escudero-Lara et al., 2020). This pattern demonstrates that in some disease contexts, titrated chronic THC can provide both symptomatic and disease-modifying benefits.

In cisplatin-induced neuropathic cancer pain models, THC (10 and 20 mg/kg i.p.) reduced mechanical hypersensitivity, but 5 mg/kg was ineffective, and chronic daily THC (6 or 10 mg/kg i.p.) acutely reversed allodynia, with females developing tolerance to its anti-allodynic effects faster than males (Henderson-Redmond et al., 2021). In paclitaxel-treated mice, THC (0.625–20 mg/kg i.p.) given before each chemotherapy dose attenuated mechanical hypersensitivity on day 9 at several doses and remained effective at 10–20 mg/kg on day 14 (King et al., 2017) although another study using daily THC (5–10 mg/kg i.p. for 8–9 days) found that tolerance to antinociception, motor impairment, and hypothermia emerged within 4–8 days and that rimonabant precipitated withdrawal (paw tremors, headshakes, scratching), indicating CB_1_R-dependent dependence (Deng et al., 2015).

In pain-naïve mice, chronic THC (twice daily for 6.5 days at 10 mg/kg or at escalating 10, 20, 30 or 10, 30, 60 mg/kg i.p.) produced dose- and region-dependent tolerance across antinociception, hypothermia, locomotor suppression, and ring immobility. Acute THC then generated near-maximal tail-flick analgesia in vehicle-treated but only blunted effects in THC-pre-exposed mice (McKinney et al., 2008). Another study giving daily THC (0.3–10 mg/kg i.p.) to naïve mice observed little change in tail-flick nociception, with 10 mg/kg approaching significance on day 14 but not day 20, again illustrating that analgesic tolerance and baseline pain state strongly condition THC’s observable effects (Long et al., 2010). Chronic THC vapor inhalation also altered midbrain vlPAG intrinsic and synaptic properties, reducing synaptic inhibition and firing in males and rescuing CFA-induced increased inhibition in females, implying sex- and circuit-specific adaptations to long-term inhaled THC exposure (Kelley et al., 2025).

Overall, acute THC acts as an analgesic across inflammatory, neuropathic, visceral, and chemotherapy-induced pain models (English et al., 2024; Javadi-Paydar et al., 2018; Jenkins et al., 2025; Harris et al., 2019; Craft et al., 2023; Mitchell et al., 2021; Casey et al., 2017; Pearl-Dowler et al., 2023; Henderson-Redmond et al., 2021; King et al., 2017; Deng et al., 2015; Morris et al., 2024). However, its analgesic doses nearly overlap with CB_1_R-mediated psychoactivity and motor impairment, resulting in a narrow therapeutic window. Across systemic routes of administration, the coupling between analgesia and central nervous system (CNS) side effects only slightly improves in some neuropathic models, whereas intrathecal delivery can partially dissociate spinal anti-hyperalgesia from canonical cannabinoid tetrad behaviors. Drug-route interactions are one way to increase tolerability (Gadotti et al., 2023; Casey et al., 2022, 2017). Sex, pain state, and disease context also contribute to THC’s efficacy and tolerance, with females in some chemotherapy-induced neuropathy models developing anti-allodynic tolerance more rapidly, and certain chronic or disease-specific paradigms (for example, oral self-administration or endometriosis) supporting chronic analgesia and potential disease-modifying effects (Jenkins et al., 2025; Mabou Tagne et al., 2024; Abraham et al., 2020; Escudero-Lara et al., 2020; Henderson-Redmond et al., 2021; King et al., 2017; Deng et al., 2015; Kelley et al., 2025). Collectively, these data position THC as a reliable preclinical analgesic, though limited by psychoactive liability and tolerance, motivating strategies that widen its therapeutic index like targeted spinal delivery, disease-tailored dosing schedules, and rational polytherapy with other cannabinoids, terpenes, or non-cannabinoid analgesics.

#### 2.1.2 CBD

Across preclinical models, CBD shows limited and variable acute antinociception but more consistent efficacy with subchronic or chronic dosing, particularly in neuropathic and chemotherapy-induced pain. In pain-naïve rodents, acute CBD often fails to alter tail-flick or hot-plate thresholds: for example, i.p. CBD (10–100 mg/kg) did not increase tail-withdrawal latency in rats and daily CBD (1–50 mg/kg i.p. for 3 weeks) produced modest hyperthermia and anxiolytic-like behavior without detectable changes in nociception (Varvel et al., 2006; Morris et al., 2024).

In a CCI neuropathic model, acute oral CBD reduced mechanical and cold allodynia but only at relatively high doses, with ED50s of 51 and 38 mg/kg and no cannabimimetic side effects (Mitchell et al., 2021). Route of administration strongly shapes efficacy. For example: s.c. CBD alleviated CCI-induced mechanical allodynia with ED_50_s of 3.6–3.9 mg/kg and no cannabimimetic behaviors whereas intrathecal CBD dose-dependently reduced CCI-evoked mechanical and cold allodynia with ED_50_s of 21 and 11 nmol and, again, without cannabis-like side effects (Casey et al., 2022, 2017). In a spinal nerve ligation model, intrathecal CBD (10–50 μg) modestly increased paw-withdrawal thresholds and latencies, confirming a spinal analgesic effect at microgram doses, though less robust than that of the modified analog dihydroxyl-CBD (Xiong et al., 2012).

Subchronic systemic CBD regimens support its utility in established neuropathic pain. In CCI mice, low doses of CBD (0.3, 3, 10, or 30 mg/kg i.p. once daily for 3 days, beginning 22 days after surgery) reversed mechanical and thermal allodynia, reduced cold hyperalgesia, and improved anxiety-like behavior across all doses tested (Silva-Cardoso et al., 2021). A follow-up study showed that the same dosing range (0.3–10 mg/kg i.p. for 3 days) reversed “affective” pain aversion in a conditioned place preference paradigm and restored mechanical and thermal thresholds in CCI animals (Silva-Cardoso et al., 2023). In an SNL model, CBD given i.p. at 5, 10, and 30 mg/kg improved von Frey thresholds, acetone responses, and tail-flick latency, and 5 mg/kg additionally alleviated depressive-like behavior, although higher doses were not tested for all endpoints (Shen et al., 2024).

Chronic systemic CBD appears especially effective in neuropathic models with affective comorbidity. In selectively bred Carioca rats (high-anxiety, low-anxiety, and control lineages), daily CBD (5 mg/kg i.p., days 14–23) after sciatic constriction injury reduced mechanical allodynia across all lineages, although less so in high-anxiety rats, and had lineage- and pain-state–dependent anxiolytic effects (Macêdo-Souza et al., 2026). In another sciatic constriction model, CBD (20 mg/kg i.p.) produced antinociception, and the effect of a lower CBD dose (2 mg/kg) was potentiated by the aminopeptidase-N inhibitor bestatin. Local delivery of a non-selective opioid receptor antagonist, naloxone, reversed CBD-induced analgesia. μ- and δ- antagonists, CTOP and naltrinole, but not the κ-, opioid antagonist, norBNI, partially reversed CBD’s analgesia. This implicates peripheral endogenous opioids and/or receptors in CBD’s mechanism (de Almeida et al., 2025).

CBD shows strong prophylactic actions in chemotherapy-induced peripheral neuropathy (CIPN). In a rat paclitaxel model, co-administration of CBD (5 mg/kg) with each chemotherapy dose prevented the development of mechanical allodynia and cold hyperalgesia, keeping sensory thresholds at baseline while vehicle-treated animals developed CIPN (Ippolito et al., 2025). This protection persisted for at least 7 weeks after treatment, and high-speed videography confirmed that CBD blocked both allodynic responses to innocuous stimuli and exaggerated responses to noxious stimuli (Ippolito et al., 2025). Earlier work in a mouse paclitaxel model similarly showed that CBD (2.5–10 mg/kg i.p.) given during chemotherapy prevented the onset of mechanical allodynia via a 5-HT1A-dependent mechanism, without producing rewards, cognitive deficits, or interfering with antitumor efficacy (Ward et al., 2014). By contrast, in cisplatin-induced neuropathic pain, CBD (5, 10, or 20 mg/kg i.p.) produced little change in mechanical hypersensitivity, highlighting that CBD’s chemotherapy-related benefits are model dependent (Sepulveda et al., 2022).

In carrageenan-induced inflammatory pain, oral CBD (10–100 mg/kg) alone did not provide clear antinociception but reduced inflammation in males (Jenkins et al., 2025), suggesting a dissociation between anti-inflammatory and reflexive pain-suppressive actions. In pain naïve rats, CBD-dominant vapor (100–400 mg/mL) induced hypothermia and suppressed locomotor activity without altering tail-withdrawal latency (Javadi-Paydar et al., 2019). In a CFA inflammatory pain model, acute exposure to vaporized CBD-dominant cannabis extract (200 mg/mL) likewise failed to affect mechanical allodynia in either sex; only repeated twice-daily exposure reduced allodynia, and exclusively in males at 100 and 200 mg/mL (Craft et al., 2023).

Collectively, these findings demonstrate that CBD produces limited acute antinociception in pain naïve and inflammatory pain models, and that it has more reliable efficacy with repeated dosing in neuropathic and chemotherapy-induced pain states. Across neuropathic pain models, CBD attenuates mechanical and cold allodynia, improves affective-motivational and anxiety-like behaviors, and can engage peripheral opioid mechanisms without cannabimimetic side effects (Casey et al., 2022; Mitchell et al., 2021; Casey et al., 2017; Xiong et al., 2012; Silva-Cardoso et al., 2021, 2023; Shen et al., 2024; Macêdo-Souza et al., 2026; de Almeida et al., 2025). Lastly, prophylactic administration during paclitaxel treatment does prevent CIPN-like hypersensitivity while conserving antitumor actions, although similar protection is not observed in cisplatin models. Thus, these studies emphasize the importance of closely considering the context- and model-specific constraints on CBD’s analgesic potential (Ippolito et al., 2025; Ward et al., 2014; Sepulveda et al., 2022).

Cannabidiolic acid (CBDA), the carboxylated precursor of CBD present in raw, unheated *Cannabis*, has been comparatively understudied for analgesic potential relative to CBD. In a carrageenan-induced inflammatory pain model, CBDA reduced hyperalgesia and edema at lower doses than those required for comparable effects with THC. This suggests higher potency at inflammatory pain targets (Rock et al., 2018). CBDA is a more potent inhibitor of COX-2 than CBD (Takeda et al., 2008) and shows greater efficacy at 5-HT1A receptors than CBD in models of nausea and vomiting (Bolognini et al., 2013), targets already implicated in phytocannabinoid analgesia more broadly. Because CBDA is the predominant cannabinoid in raw, unheated plant material and is usually retained in part in commercially available full-spectrum extracts, its unique contribution to analgesia is relevant to *Cannabis*-based pain products. However, dedicated preclinical studies of CBDA in chronic or neuropathic pain models remain sparse, representing an important gap for future research.

#### 2.1.3 Minor cannabinoids

In addition to CBD and THC, *Cannabis* contains many minor phytocannabinoids that are comparatively understudied. Cannabigerol (CBG), is a non-intoxicating precursor of THC and CBD has anti-inflammatory, antimicrobial, and neuroprotective activity in preclinical models (Li et al., 2024). Cannabinol (CBN), is an oxidative degradation product of THC and acts as a partial agonist at both CB_1_R and CB_2_R. Studies have demonstrated sedative, anti-inflammatory, antibacterial, and analgesic effects (Khouchlaa et al., 2024). Cannabichromene (CBC) acts at CB_2_Rs over CB_1_Rs and has shown anti-inflammatory, antinociceptive, antibacterial, and anticonvulsant properties in animal models (Sepulveda et al., 2024). Cannabivarin (CBV), an analog of CBN, remains one of the least-characterized phytocannabinoids, with no dedicated pharmacological studies published to date. Cannabielsoin (CBE), an oxidation product of CBD, has recently been shown to act as a biased CB_1_R agonist that preferentially engages G_*i*_/cAMP signaling over β-arrestin recruitment (Haghdoost et al., 2024). Cannabicitran (CBT), a cannabinoid biosynthesized from CBDA, has been reported in early work to lower intraocular pressure, suggesting possible relevance to glaucoma (Filipiuc et al., 2021).

#### 2.1.4 Terpenes

In this review, we focus on a handful of the most-well studied terpenes in the context of pain assays and their emerging relevance to Cannabis-based analgesia. These terpenes were selected based on robust preclinical evidence for antinociceptive and anti-inflammatory effects across various pain models. These terpenes also rank among the most abundant in commercially available Cannabis. Analyses of retail products consistently identify myrcene, β-caryophyllene, limonene, and linalool as the dominant terpene chemotypes (Fischedick, 2017; Smith et al., 2022). Together, they demonstrate how individual terpenes can engage complementary mechanisms to phytocannabinoids, including spinal adenosinergic signaling, CB_2_R-mediated immunomodulation (CB_2_R being predominantly expressed by immune cells), peripheral anti-inflammatory actions, and supraspinal modulation of nociceptive processing. This supports the hypothesis that terpenes are not only flavor/aromatic constituents but pharmacologically active contributors to the analgesic profile of cannabis preparations (Liktor-Busa et al., 2021). For a thorough review of terpene pharmacokinetics, receptor targets, and broader pharmacology beyond the scope of this section, see Liktor-Busa et al., 2021.

##### 2.1.4.1 Linalool

Linalool’s primary botanical source, lavender (*Lavandula angustifolia*), has a centuries-long history of use as an analgesic and anxiolytic in traditional medicine, and recent clinical and preclinical work supports biological plausibility for these effects (Civilyte et al., 2025). In acute visceral pain, s.c. (±)-linalool (25–75 mg/kg) dose-dependently reduced acetic acid–induced writhing, which was reversed by naloxone and atropine, implicating opioidergic and muscarinic cholinergic mechanisms, while the (-)-enantiomer administered i.p. (25–150 mg/kg) produced dose-dependent antinociception in the formalin test via adenosine and opioid pathways, with hot plate analgesia only at the highest dose (100 mg/kg) via adenosine A1/A2a receptor signaling (Peana et al., 2003, 2004a,^2006^). S.c. administration at 50–200 mg/kg also dose-dependently prevented carrageenan-induced hyperalgesia and reversed glutamate- and prostaglandin E2–evoked hyperalgesia (Peana et al., 2004a,b). In another study, i.p., oral, and intrathecally administered linalool dose-dependently inhibited glutamate-induced paw licking and blocked spinal nociceptive behaviors (Patrícia Aparecida Batista et al., 2008). Intraplantar linalool (5–10 μg/paw) locally reversed paclitaxel-induced mechanical allodynia and hyperalgesia through peripheral opioid receptors and similarly attenuated capsaicin-, glutamate-, and formalin-induced nociception via local mu/kappa-opioid receptor activation (Katsuyama et al., 2015, 2012; Sakurada et al., 2011). In formaldehyde- and CFA-induced arthritis in rats, oral linalool (25–75 mg/kg) dose-dependently reduced paw licking and paw edema, inhibited arthritic histopathology, and attenuated oxidative stress markers, with the 75 mg/kg dose combined with indomethacin producing greater joint protection than either alone (Riaz et al., 2025).

In neuropathic pain, a single acute dose of 50 mg/kg i.p. did not attenuate mechanical or thermal sensitivity in a SNL model, whereas with a week of repeated dosing (50 mg/kg × 2 daily for 10 days) transiently attenuated mechanical allodynia (Berliocchi et al., 2009). In other studies, however, both acute and repeated systemic dosing (50–200 mg/kg, i.p.) reduced mechanical and thermal hypersensitivity and paw edema in a CFA model (Patricia Aparecida Batista et al., 2010). In a recent study examining CIPN and lipopolysaccharide (LPS)-induced inflammatory pain in both sexes, i.p. linalool (200 mg/kg) produced antinociception comparable to morphine in both models without conditioned place preference, and sub-analgesic doses synergized with morphine (Schwarz et al., 2024). Spinal A2aR antagonism and CRISPR-mediated knockdown partially attenuated linalool antinociception in CIPN, suggesting a spinal adenosinergic mechanism that partially overlaps with that of other cannabis terpenes (Schwarz et al., 2024). Peripherally injected linalool (intraplantar, 0.25–1 mg/paw) also transiently attenuated allodynia and suppressed spinal ERK phosphorylation in animals in pSNL-induced neuropathic pain (Kuwahata et al., 2013). In a Freund’s adjuvant-induced chronic synovitis model in rats, oral linalool (100–400 mg/kg) given for 28 days dose-dependently reduced paw volume and joint inflammation while suppressing serum IL-17 and normalizing spleen and thymus indices, with effects comparable to indomethacin, suggesting that linalool modulates the adaptive immune component of joint inflammation in addition to its acute anti-inflammatory actions (Nawaz et al., 2023).

Acute and chronic inhalation of linalool-dominant lavender essential oil (0.1%) increased mechanical and thermal thresholds in CFA mice via olfactory-piriform-insular GABAergic circuits (Yang et al., 2024). In addition, a separate study found that acute inhalation of lavender essential oil reduced hyperalgesia in formalin and CCI models via opioid and CB_1_R-dependent mechanisms (Donatello et al., 2020). Acute inhalation of linalool vapor alone reduced formalin and hot-plate pain behaviors and requires intact hypothalamic orexin neuron functioning (Tashiro et al., 2016). In a fibromyalgia-like chronic muscle pain model, oral linalool (25–100 mg/kg) reduced mechanical hyperalgesia without motor impairment, with a β-cyclodextrin formulation enhancing efficacy (Nascimento et al., 2014). In a separate post-operative pain and a reserpine-induced fibromyalgia-like model, linalool (200 mg/kg, i.p.) reduced mechanical hypersensitivity in both sexes via A2aR-dependent mechanisms, with antinociception blocked by the selective A2aR antagonist istradefylline, identifying a conserved spinal adenosinergic mechanism for linalool across multiple chronic pain states (Seekins et al., 2025). Taken together, these studies show that linalool has antinociceptive efficacy across visceral, inflammatory, arthritic, and chemotherapy-induced pain models, with neuropathic pain requiring repeated dosing or local delivery, and a conserved spinal adenosinergic mechanism emerging as a consistent mechanism across many chronic pain states.

##### 2.1.4.2 β-Caryophyllene

β-Caryophyllene (BCP) is a bicyclic sesquiterpene found in spices like black pepper, cloves, as well as many *cannabis* cultivars. Notably, it’s the only terpene known to act as a selective CB_2_R agonist, which underlies its analgesic/anti-inflammatory properties. BCP is unique in the literature due to the fact that it’s commonly tested orally, likely reflecting status as an FDA-approved food additive and its translational relevance as a dietary compound, despite oral bioavailability being limited in preclinical models. In acute visceral and inflammatory pain, 25–50 mg/kg oral BCP reduced formalin-induced pain and carrageenan paw edema via CB_2_R (Klauke et al., 2014). Similarly, intraplantar BCP (2.25–18 μg/paw) dose-dependently attenuated capsaicin-induced nociception via peripheral CB_2_R and local mu-opioid receptor activation with sub-effective doses actually enhancing morphine’s antinociceptive effects (Katsuyama et al., 2013). Orally delivered low doses of BCP (1–10 mg/kg) dose-dependently reduced acute thermal pain and formalin-evoked nociception that are blocked by both opioid and CB_2_R antagonist (naloxone and AM630 respectively) (Paula-Freire et al., 2014). In postsurgical pain, 10–75 mg/kg i.p. BCP results in dose- and time- dependent reduction of incision -induced mechanical hyperalgesia (Klawitter et al., 2024). This is associated with a ∼3x increase in 2-AG levels via MAGL inhibition, implicating an eCB-mediated analgesia via secondary CB_1_R and CB_2_R activation (Klawitter et al., 2024).

In neuropathic pain, acute and chronic oral BCP (25 mg/kg, ×2 daily) successfully reversed paclitaxel-induced mechanical and cold allodynia in a CB_2_R-dependent manner (Segat et al., 2017). It is also a prophylactic agent, preventing CIPN and spinal microglial activation and IL-1β (Segat et al., 2017). High doses of chronic oral BCP (50–100 mg/kg) reduced mechanical allodynia and thermal hyperalgesia in a streptozotocin-induced diabetic neuropathy via PPARγ upregulation and spinal cytokine modulation, while acute BCP (40 mg/kg, i.p.) reversed mechanical allodynia in antiretroviral-induced neuropathy through CB_2_R-selective mechanisms without motor impairment (Aguilar-Ávila et al., 2019; Aly et al., 2019). In a CCI mode, chronic oral BCP attenuated thermal hyperalgesia and mechanical allodynia, reduced spinal microglial activation and proinflammatory cytokines in a CB_2_R-dependent manner (Klauke et al., 2014). Acute BCP (10–100 mg/kg, i.p.) reduced mechanical allodynia in a CCI model via CB_2_R (Bilbrey et al., 2022). In chronic inflammatory arthritis, daily oral BCP (10 mg/kg for 14 days) reduced clinical severity scores, joint swelling, cartilage damage, and pro-inflammatory cytokines in a collagen antibody–induced arthritis model via CB_2_R–PPARγ crosstalk (Irrera et al., 2019). Chronic pretreatment (400 mg/kg, daily for 7 days) reduced mechanical hypersensitivity and joint inflammation in MSU crystal–induced gouty arthritis through NLRP3 inflammasome and NF-κB suppression (Li et al., 2021). Collectively, BCP demonstrates reliable antinociception across acute inflammatory, postsurgical, chemotherapy-induced, diabetic, and CCI neuropathic pain models, primarily via CB_2_R activation.

##### 2.1.4.3 Myrcene

Myrcene is a monocyclic monoterpene, found commonly in hops, mangoes, and is one of the most abundant terpenes in *Cannabis* (Fischedick, 2017; Smith et al., 2022). One of the first studies to show its analgesic properties demonstrated that in acute thermal and visceral pain, 10–40 mg/kg i.p. and s.c. administration of β-myrcene inhibited nociception in hot-plate and acetic acid writhing tests, with antinociception blocked by naloxone and yohimbine, implicating endogenous opioid and α2-adrenoceptor pathways (Rao et al., 1990). Oral myrcene (5–10 mg/kg) similarly increased hot-plate latency for over 4 h and reduced formalin-evoked licking in both neurogenic and inflammatory phases, again reversed by naloxone, further supporting opioid system involvement in its acute antinociceptive effects (Paula-Freire et al., 2013). In acute inflammatory hyperalgesia, myrcene produced dose-dependent peripheral analgesia by reducing carrageenan- and prostaglandin E2-induced hyperalgesia without affecting dibutyryl cyclic adenosine monophosphate (dbcAMP)-induced pain, suggesting a peripheral site of action distinct from aspirin-like drugs, and without development of tolerance unlike morphine (Lorenzetti et al., 1991).

In models of neuropathic pain, 5–10 mg/kg of oral myrcene reduced mechanical and thermal hyperalgesia in a CCI model over 14 days, with efficacy increasing across the treatment period, though without reducing sciatic nerve IL-1β in contrast to eugenol which was tested alongside it (Paula-Freire et al., 2016). Acute myrcene (1–200 mg/kg i.p.) also dose-dependently increased mechanical nociceptive thresholds in a nerve injury model without sedative or hypothermic effects, with antiallodynic effects inhibited by a CB_1_R antagonist, suggesting indirect CB_1_R pathway involvement despite an apparent lack of direct receptor activation (Alayoubi et al., 2025). The most parsimonious explanation is that myrcene elevates endocannabinoid tone which then activates CB_1_R to produce analgesia, rather than binding CB_1_R directly. *In vitro* TRUPATH assays confirmed myrcene neither activates CB_1_R nor modulates its response to exogenous or endogenous agonists (Alayoubi et al., 2025). In summary, myrcene shows analgesic efficacy in acute thermal, visceral, and inflammatory pain models as well as in chronic neuropathic pain.

##### 2.1.4.4 Limonene

Limonene is a cyclic monoterpene and one of the most abundant terpenes found in citrus and is found in numerous *cannabis* cultivars. In acute visceral and inflammatory pain, 25–50 mg/kg i.p. limonene reduced acetic acid-induced writhing and inhibited the late-phase of the formalin test. This treatment did not affect hot plate latency and also wasn’t reversed by naloxone, suggesting a peripheral, non-opioid mechanism (do Amaral et al., 2007). Oral limonene similarly inhibited the late phase of formalin-evoked nociception and reduced leukocyte migration, cytokine production, and protein extravasation in a carrageenan air-pouch model, with effects comparable to those of limonene-rich citrus essential oils tested as well (Amorim et al., 2016). An acute 10 mg/kg oral dose of limonene also reduced mechanical hypersensitivity induced by intrathecal gp120, TNF-α, and IL-1β, and prevented gp120-evoked increases in serum IL-1β while enhancing spinal antioxidant enzyme expression, suggesting central anti-inflammatory and analgesic actions (Piccinelli et al., 2017).

In a chronic neuropathic pain model, 10 mg/kg oral limonene treatment daily for 15 days significantly reduced SNI-induced mechanical hyperalgesia and depressive-like behavior without affecting locomotor activity, though it did not attenuate cold hyperalgesia at the final timepoint (Piccinelli et al., 2015). In a fibromyalgia-like chronic musculoskeletal pain model, 50 mg/kg oral limonene–β-cyclodextrin complex produced more robust and sustained anti-hyperalgesia than uncomplexed (plain limonene dissolved in vehicle, without cyclodextrin encapsulation) limonene, reduced dorsal horn Fos expression, and attenuated capsaicin-induced nociception via spinal GABAergic mechanisms rather than opioid pathways, with flumazenil but not naloxone reversing its effects (Araújo-Filho et al., 2017). Intraplantar limonene caused acute TRPA1-mediated nociception while systemic limonene reduced TRPA1-dependent H_2_O_2_-induced nociceptive behaviors, indicating that route of administration determines whether limonene acts as a nociceptive or antinociceptive agent at this channel (Kaimoto et al., 2016). A related compound, (+)-limonene epoxide, a metabolic oxidation product of limonene, also produced dose-dependent antinociception across acetic acid writhing, both phases of the formalin test, and thermal nociception, with effects reversed by naloxone, indicating opioid mechanism involvement, and additionally reduced carrageenan paw edema and leukocyte migration (de Almeida et al., 2017). Unlike the parent compound, the opioid-sensitive mechanism of limonene epoxide suggests that oxidative metabolism may alter the pharmacological profile of limonene. In summary, limonene is a modestly efficacious analgesic terpene with anti-inflammatory and mood-stabilizing properties, particularly relevant to pain conditions with affective comorbidity. However, its analgesic potency appears lower than that of linalool or β-caryophyllene at comparable doses.

##### 2.1.4.5 α-Terpineol

α-Terpineol is a monocyclic monoterpenoid alcohol found in eucalyptus, tea tree, and numerous other aromatic plants, and is present in some *cannabis* cultivars. In acute pain, 25–100 mg/kg i.p. α-terpineol shows dose-dependent antinociception across acetic acid writhing, both phases of the formalin test, glutamate- and capsaicin-induced paw licking, and hot-plate tests, with central effects at 100 mg/kg comparable to morphine and no motor impairment, suggesting both peripheral and supraspinal analgesic mechanisms (Quintans-Júnior et al., 2011). In acute inflammatory hyperalgesia, the same dose range dose-dependently attenuated carrageenan-, TNF-α-, PGE_2_-, and dopamine-induced mechanical hyperalgesia, inhibited neutrophil influx in carrageenan pleurisy, and reduced macrophage nitrite production, indicating broad peripheral anti-inflammatory activity via cytokine cascade inhibition and nitrous oxide (NO) pathway modulation (de Oliveira et al., 2012). In the formalin test, i.p. α-terpineol (40–80 mg/kg) produced antinociception in both formalin phases comparable to morphine and ketorolac, with effects reversed by naloxone, L-NAME, methylene blue, and glibenclamide, implicating opioid receptors and the NO/cGMP/KATP channel pathway as contributing mechanisms (Safaripour et al., 2018). In chronic neuropathic pain, once-daily α-terpineol (25–100 mg/kg, i.p.) for 14 days dose-dependently reduced CCI-induced mechanical allodynia, cold allodynia, and thermal hyperalgesia, with 100 mg/kg efficacy matching gabapentin, and additionally reduced spinal microglial activation and IL-1β/TNF-α concentrations more effectively than gabapentin (Soleimani et al., 2019). A single acute i.p. dose also reduced mechanical allodynia in a CCI neuropathic pain model in a CB_1_R/CB_2_R-sensitive manner, placing it alongside BCP as a terpene with cannabinoid receptor-relevant antinociceptive activity (Bilbrey et al., 2022). Overall, α-terpineol demonstrates acute antinociception across multiple pain modalities and reliable efficacy in chronic neuropathic pain with repeated dosing, engaging peripheral anti-inflammatory, NO/cGMP, and cannabinoid systems.

##### 2.1.4.6 α-Bisabolol

α-Bisabolol is a sesquiterpene alcohol found in German chamomile and several other aromatic plants, and has emerging relevance to *cannabis* research as a minor terpene constituent of some cultivars. In acute visceral and inflammatory pain, oral α-bisabolol (50–200 mg/kg) dose-dependently reduced acetic acid–induced writhing, formalin phase 2 licking, and carrageenan- and dextran-induced paw edema, inhibited leukocyte migration, TNF-α production, and neutrophil degranulation, and had no effect on hot-plate latency, suggesting primarily peripheral anti-inflammatory and antinociceptive actions (Rocha et al., 2011; Leite Gde et al., 2011). In a visceral nociception model, oral α-bisabolol suppressed abdominal constrictions induced by acetic acid, capsaicin, formalin, and mustard oil without motor impairment, with mustard oil antinociception resistant to KATP, NO, α_2_-adrenergic, and 5-HT_3_ antagonists but showing additivity with a TRPV1 blocker, implicating TRPV1-related mechanisms in its visceral analgesic effects (de Oliveira et al., 2012). When combined with diclofenac in fixed-ratio combinations, oral α-bisabolol produced synergistic antinociception and anti-inflammatory effects while significantly reducing diclofenac-induced gastric damage, suggesting potential as an adjunct to reduce NSAID-associated gastrointestinal toxicity (Ortiz et al., 2018).

In chronic pain, oral α-bisabolol (50 mg/kg) and a β-cyclodextrin complex with enhanced efficacy both reduced mechanical and thermal hyperalgesia in CFA inflammatory and partial sciatic nerve ligation neuropathic pain models, decreased spinal TNF-α and microglial activation, and increased IL-10, with the cyclodextrin formulation again outperforming free compound (Fontinele et al., 2019). In trigeminal neuropathic pain, acute α-bisabolol (30 mg/kg, i.p.) reversed infraorbital nerve injury–induced facial mechanical hypersensitivity and attenuated central sensitization of medullary dorsal horn neurons, demonstrating efficacy in a craniofacial neuropathic pain model with both behavioral and electrophysiological endpoints (Melo et al., 2019). Collectively, these studies show that α-bisabolol demonstrates consistent antinociceptive and anti-inflammatory efficacy across acute visceral, inflammatory, and chronic neuropathic pain models primarily via peripheral mechanisms, with β-cyclodextrin complexation enhancing its analgesic profile similarly to what has been observed with linalool and limonene.

#### 2.1.5 Entourage

While individual cannabinoids or terpenes produce distinct pharmacology effects, oral cannabinoid-rich extracts frequently show enhanced or prolonged efficacy compared with matched doses or isolated compounds. This suggests that interactions among multiple phytocannabinoids and/or terpenes, the “entourage effect”, may contribute to improved therapeutic outcomes. This is potentially therapeutically useful given that chronic THC alone is constrained by tolerance, withdrawal, and CB_1_R-mediated side effects with daily dosing (Abraham et al., 2020; McKinney et al., 2008). In a CFA inflammatory pain model, a non-euphoric phytocannabinoid elixir (NEPE14) reduced thermal and mechanical hyperalgesia after i.p. (6.6–20.7 mL/kg) or oral-mucosal (30–300 μL) dosing, yet the same range did not alter tail-withdrawal latency in pain-naïve rats, indicating context-dependent analgesia without generalized sedation (Morris et al., 2024).

THC:CBD combinations exhibit route- and ratio-dependent interactions. An oral 1:1 THC:CBD mixture by weight (≈8:1 by ED_50_) produced a dose-dependent reduction in CCI allodynia but no synergy and generated synergistic side effects that increased with the CBD proportion (Mitchell et al., 2021). In contrast, s.c. THC + CBD in CCI produced synergistic anti-allodynia at low total doses (≈200-fold potency increase) without cannabinoid-like side effects, whereas higher doses achieved greater efficacy accompanied by THC-like adverse effects (Casey et al., 2017). At the spinal level, a 1:1 intrathecal THC:CBD combination synergistically reduced CCI-induced mechanical and cold allodynia, with roughly twofold higher potency than predicted for additivity and no detectable side effects (Casey et al., 2022).

Pharmacokinetic entourage effects are also evident but are highly dose- and route-dependent. When high-dose CBD (30 mg/kg i.v.) was given 10 min before a threshold THC dose (0.3 mg/kg i.v.), it potentiated tail-flick antinociception and elevated THC blood and brain concentration, likely via CYP450-mediated inhibition of THC metabolism. However, CBD at or below maximally effective THC dose (3 mg/kg) failed to alter any tetrad measure, even 30 mg/kg i.v. CBD did not potentiate the antinociceptive effects of inhaled marijuana smoke despite producing equivalent blood CBD levels (Varvel et al., 2006). These findings suggest that pharmacokinetic THC-CBD interactions depend on the timing and route of co-administration. In a mouse migraine model, a high-CBD:low-THC ratio (100:1; 100 mg/kg CBD + 1 mg/kg THC) rescued photophobia and cortical spreading depolarization–induced grimace, with partial rescue of squint in males, effects not observed with either CBD or THC alone (Zorrilla et al., 2025).

In neuropathic male mice, mechanical hypersensitivity decreased after 6 mg/kg i.p. THC:CBD only at a 2:1 ratio, whereas females showed reductions at 1:2 and 2:1 ratios; when equivalent doses were delivered as plant extracts, males gained additional benefit from adding THC to CBD extract (1:2, 1:1, 2:1 vs. 0:1), but females did not, suggesting sex-specific actions and a ceiling effect of CBD in females (Borgonetti et al., 2023). Not all combinations are advantageous. In a carrageenan-induced edema model, oral CBD attenuated THC’s antinociceptive effects such that THC + CBD was sub-additive relative to THC alone, and isobolographic and dose-addition analyses confirmed that THC monotherapy was superior for analgesia (Jenkins et al., 2025).

Chemotherapy-related pain models reveal both synergistic and additive actions of CBD:THC ratios. In paclitaxel-induced neuropathic pain, a 1:1 CBD + THC i.p. regimen produced a biphasic, dose-related attenuation of mechanical sensitivity, with very low individually ineffective doses of each cannabinoid becoming synergistic when combined (King et al., 2017). In vapor inhalation studies, a 1:4 THC:CBD mixture (25 mg/mL THC + 100 mg/mL CBD) and a higher-dose 100:400 mg/mL combination decreased temperature and activity in an approximately additive manner, although pain behavior was not assessed (Javadi-Paydar et al., 2018).

Whole-plant and broad-spectrum cannabis extracts further support the concept of entourage-like analgesia enhancement. Full-extract *Cannabis* containing cannabinoids and terpenes (4.8, 9.7, 14.5 mg/kg i.p.) increased hot-plate and tail-flick latencies and reduced acetic-acid–induced writhing, whereas a terpene-reduced extract (4.2, 8.4, 12.7 mg/kg, i.p.) increased tail-flick latency and reduced writhing but failed to increase hot-plate latency at the lowest dose, implying that terpenes selectively enhance some supraspinal nociceptive endpoints (Harris et al., 2019). A broad-spectrum cannabis oil (CBD:THC ≈ 11:1; 40% total cannabinoids) reversed reserpine-induced mechanical hyperalgesia when given orally, intraplantar, intrathecally, or intracerebroventricular and chronic oral dosing (1 mg/kg for 10 days) improved mechanical and heat sensitivity and reduced depressive-like behaviors without inducing locomotor impairment, highlighting route-dependent yet behaviorally favorable actions (Ferrarini et al., 2022). Finally, a CBD-enriched *Cannabis* extract (eCBD; 64.5% CBD, 4% THC plus minor cannabinoids, terpenes, and flavonoids) administered orally for 1 week beginning day 7 after CCI fully reversed thermal hyperalgesia and partially reversed mechanical allodynia, outperforming equivalent doses of pure CBD or THC and providing strong preclinical evidence for entourage-like enhancement in neuropathic pain (Comelli et al., 2008). These studies establish that whole-cannabis preparations can in some cases outperform cannabinoids as single agents, though the mechanistic basis of this is not always pharmacodynamic. An important factor to consider is the metabolic interaction between CBD and THC. CBD is an inhibitor of cytochrome P450 enzymes (i.e., CYP3A4 and CYP2C9) which are primary metabolic routes for THC (Yamaori et al., 2011; Bansal et al., 2023). Therefore, co-administration may reduce first-pass clearance of THC, thereby increasing its systemic and central bioavailability and prolonging its half-life. CYP450 inhibition paired with increased antinociception has been observed (Comelli et al., 2008), suggesting that what looks like pharmacodynamic synergy in whole-plant preparations might simply be explained by CBD-driven increases in THC exposure. Table 1 summarizes the key preclinical findings reviewed above, organized by cannabinoid/terpene compound, pain model, dose, route, and analgesic outcome.

**TABLE 1:** Preclinical studies on the analgesic effects of phytocannabinoids and terpenes.

Compound	Dose	Route	Regimen	Pain type	Outcome	References
THC						
Δ9-THC	Moderate-High (1.8–30 mg/kg)	Systemic	Acute	Naïve/Thermal	↑ tail-withdrawal and hot-plate latency; cannabimimetic side effects at higher doses	Morris et al., 2024; English et al., 2024; Javadi-Paydar et al., 2018; Harris et al., 2019
Δ9-THC	Low–Moderate (1–10 mg/kg i.p./oral; 200–400 mg/mL vapor)	Systemic/Vapor	Acute	Inflammatory	↓ thermal hyperalgesia and mechanical allodynia; sex-dependent effects; selective tolerance with repeated vapor	Jenkins et al., 2025; Harris et al., 2019; Craft et al., 2023
Δ9-THC	Low–High (0.3–56 mg/kg)	Systemic/Oral	Acute	Neuropathic	↓ mechanical and cold allodynia across routes; modest therapeutic index; efficacy preserved in opioid-tolerant animals	Mitchell et al., 2021; Casey et al., 2017; Pearl-Dowler et al., 2023
Δ9-THC	Low (25 μg; ED50 14–20 nmol)	Intrathecal	Acute	Neuropathic	↓ allodynia without cannabinoid side effects; partial dissociation of spinal analgesia from central adverse effects	Gadotti et al., 2023; Casey et al., 2022
Δ9-THC	Moderate–High (0.625–20 mg/kg)	Systemic	Acute	CIPN	↓ mechanical hypersensitivity; tolerance develops within days of daily dosing; faster in females	King et al., 2017; Henderson-Redmond et al., 2021; Deng et al., 2015
Δ9-THC	Low–Moderate (0.1–5 mg/kg)	Systemic	Acute	Disease-specific	↓ mechanical and cold hypersensitivity (sickle cell); ↓ abdominal hypersensitivity (endometriosis)	Mabou Tagne et al., 2024; Escudero-Lara et al., 2020
Δ9-THC	Low–Moderate (1 mg/15 mL gelatin; 6–10 mg/kg i.p.)	Oral/Systemic	Chronic	Neuropathic (Abraham); CIPN (Henderson-Redmond)	↓ allodynia and hyperalgesia sustained over weeks; tolerance develops in some models; sex differences in tolerance rate	Abraham et al., 2020; Henderson-Redmond et al., 2021
Δ9-THC	Low (1–3 mg/kg)	Systemic	Chronic	Disease-specific	↓ hypersensitivity and disease markers without tolerance to antinociception (endometriosis); sustained mechanical analgesia (sickle cell)	Escudero-Lara et al., 2020; Mabou Tagne et al., 2024
Δ9-THC	Moderate–High (10–60 mg/kg)	Systemic	Chronic	Naïve (tolerance)	Dose-dependent tolerance to antinociception, hypothermia, and locomotion; minimal analgesic tolerance at low doses	McKinney et al., 2008; Long et al., 2010
Δ9-THC (vapor)	Moderate (exact mg/L unspecified)	Vapor	Chronic	Inflammatory	Sex-specific vlPAG circuit adaptations; ↓ inhibition in males; rescued CFA-induced inhibition in females	Kelley et al., 2025
CBD						
CBD	Moderate–High (10–100 mg/kg)	Systemic/Oral	Acute	Naïve/Inflammatory	No antinociception in naïve or inflammatory models; modest anti-inflammatory effects in males	Varvel et al., 2006; Morris et al., 2024; Jenkins et al., 2025; Javadi-Paydar et al., 2018
CBD	Low–High (oral ED50 ∼38–51 mg/kg; s.c. ED50 3.6–3.9 mg/kg; i.t. ED50 11–21 nmol)	Systemic/Oral/Intrathecal	Acute	Neuropathic	↓ mechanical and cold allodynia; oral requires high doses; s.c. and i.t. far more potent; no cannabimimetic side effects	Mitchell et al., 2021; Casey et al., 2017; Xiong et al., 2012
CBD	Low-Moderate (0.3–30 mg/kg × 3 days)	Systemic	Subchronic	Neuropathic	↓ mechanical/thermal allodynia, cold hyperalgesia, and anxiety-like behavior; reversed affective pain aversion	Silva-Cardoso et al., 2021; Silva-Cardoso et al., 2023
CBD	Low (5–10 mg/kg daily)	Systemic	Chronic	Neuropathic	↓ allodynia and hyperalgesia; improved affective and anxiety comorbidities; peripheral opioid mechanism	Shen et al., 2024; Macêdo-Souza et al., 2026; de Almeida et al., 2025
CBD	Low (Ippolito: 5 mg/kg; Ward: 2.5–10 mg/kg i.p., with each chemo dose)	Systemic	Prophylactic	CIPN (paclitaxel)	Prevented mechanical allodynia and cold hyperalgesia; protection persisted ≥ 7 weeks; no antitumor interference	Ippolito et al., 2025; Ward et al., 2014
CBD	Low–Moderate (5–20 mg/kg)	Systemic	Acute	CIPN (cisplatin)	No significant effect on mechanical hypersensitivity; model-dependent limits of CBD efficacy	Sepulveda et al., 2022
Terpenes						
Linalool	Moderate (25–75 mg/kg s.c./i.p.; 5–10 μg/paw local)	Systemic/Local/Inhalation	Acute	Visceral/Thermal	↓ writhing and formalin nociception; opioid and adenosine A1/A2a mechanisms; peripheral opioid activation (local)	Peana et al., 2003, 2004a,2004b; 2006; Batista et al., 2008; Sakurada et al., 2011; Katsuyama et al., 2012, 2015; Tashiro et al., 2016
Linalool	Moderate–High (50–200 mg/kg i.p.; 25–75 mg/kg oral (Riaz); 100–400 mg/kg oral (Nawaz, chronic); 0.1% inhalation)	Systemic/Inhalation/Oral	Acute/Chronic	Inflammatory	↓ mechanical/thermal hypersensitivity and edema; olfactory-GABAergic circuit (inhalation); β-CD complex enhances efficacy	Batista et al., 2010; Yang et al., 2024; Donatello et al., 2020; Nascimento et al., 2014; Riaz et al., 2025; Nawaz et al., 2023
Linalool	Moderate (50 mg/kg i.p.; 0.25–1 mg/paw local)	Systemic/Local	Acute/Chronic	Neuropathic	Single dose ineffective; repeated dosing transiently ↓ allodynia; local administration ↓ allodynia and spinal ERK	Berliocchi et al., 2009; Kuwahata et al., 2013
Linalool	High (200 mg/kg i.p.)	Systemic	Acute/Chronic	CIPN/Inflammatory (Schwarz); Post-operative/Fibromyalgia-like (Seekins)	↓ allodynia comparable to morphine in both sexes; synergizes with sub-analgesic morphine; spinal A2aR-dependent	Schwarz et al., 2024; Seekins et al., 2025
β-Caryophyllene	Low–Moderate (1–50 mg/kg oral; 2.25–18 μg/paw local; 10–75 mg/kg i.p.)	Oral/Local/Systemic	Acute	Inflammatory/Visceral	↓ formalin pain and edema via CB2R; potentiates morphine; postsurgical analgesia via MAGL inhibition and ↑ 2-AG	Klauke et al., 2014; Katsuyama et al., 2013; Paula-Freire et al., 2014; Klawitter et al., 2024
β-Caryophyllene	Low–Moderate (25–100 mg/kg oral; 40 mg/kg i.p.)	Oral/Systemic	Acute/Chronic	Neuropathic	↓ mechanical allodynia across neuropathic models via CB2R; prophylactic use prevented CIPN; ↓ spinal neuroinflammation	Klauke et al., 2014; Segat et al., 2017; Aguilar-Ávila et al., 2019; Aly et al., 2019; Bilbrey et al., 2022
β-Caryophyllene	Low–Moderate (10–400 mg/kg oral)	Oral	Chronic	Inflammatory arthritis	↓ joint swelling, cartilage damage, and pro-inflammatory cytokines via CB2R–PPARγ and NLRP3 suppression	Irrera et al., 2019; Li et al., 2021
β-Myrcene	Low–Moderate (1–40 mg/kg i.p./s.c.; 5–10 mg/kg oral)	Systemic/Oral	Acute/Chronic	Thermal/Visceral/Inflammatory/ Neuropathic	↓ nociception via opioid and α2-adrenoceptor pathways; peripheral anti-inflammatory without tolerance; indirect CB1R involvement in neuropathic model	Rao et al., 1990; Lorenzetti et al., 1991; Paula-Freire et al., 2013, 2016; Alayoubi et al., 2025
Limonene	Low–Moderate (25–50 mg/kg i.p.; 10–50 mg/kg oral)	Systemic/Oral	Acute/Chronic	Visceral/Inflammatory/ Neuropathic	↓ writhing and late-phase formalin (peripheral, non-opioid); ↓ SNI hyperalgesia and depressive behavior; β-CD complex enhances efficacy	do Amaral et al., 2007; Amorim et al., 2016; Piccinelli et al., 2015, 2017; Araújo-Filho et al., 2017; Kaimoto et al., 2016
(+)-Limonene epoxide	Low–Moderate (dose not specified in i.p./oral models)	Systemic	Acute	Visceral/Inflammatory/Thermal	↓ writhing, both formalin phases, and thermal nociception; ↓ carrageenan edema and leukocyte migration; opioid-sensitive (naloxone-reversible); distinct pharmacological profile from parent limonene	de Almeida et al., 2017
α-Terpineol	Moderate–High (25–100 mg/kg i.p.)	Systemic	Acute/Chronic	Thermal/Visceral/Inflammatory/ Neuropathic	↓ nociception across multiple pain types; 100 mg/kg matches gabapentin in CCI; ↓ spinal microglia and cytokines; CB1/CB2-sensitive	Quintans-Júnior et al., 2011; de Oliveira et al., 2012; Safaripour et al., 2018; Soleimani et al., 2019; Bilbrey et al., 2022
α-Bisabolol	Moderate–High (50–200 mg/kg oral; 15–30 mg/kg i.p.)	Oral/Systemic	Acute	Visceral/Inflammatory	↓ writhing and edema; peripheral anti-inflammatory action; synergistic with diclofenac while reducing GI toxicity	Rocha et al., 2011; Leite Gde et al., 2011, 2012; Ortiz et al., 2018
α-Bisabolol	Moderate (50 mg/kg oral; 30 mg/kg i.p.)	Oral/Systemic	Chronic/Acute	Neuropathic/Inflammatory	↓ mechanical/thermal hyperalgesia in CFA and pSNL; ↓ spinal neuroinflammation; reversed trigeminal neuropathic hypersensitivity	Fontinele et al., 2019; Melo et al., 2019
Entourage/Combinations						
THC + CBD	Low (synergistic) (s.c. 0.01–56 mg/kg 1:1; i.t. 1.5–153 nmol 1:1; oral 1–10 mg/kg THC)	s.c./Intrathecal/Oral	Acute	Neuropathic	s.c. and i.t.: synergistic anti-allodynia at low doses without side effects; oral: sub-additive (CBD attenuated THC)	Casey et al., 2017; Casey et al., 2022; Mitchell et al., 2021
THC + CBD	Low–Moderate (THC 1–10 mg/kg + CBD 10–100 mg/kg)	Oral	Acute	Inflammatory	Sub-additive; CBD attenuated THC antinociception; THC monotherapy superior	Jenkins et al., 2025
THC + CBD	Low–Moderate (0.04–20 mg/kg each i.p.; 100:1 CBD:THC i.p.)	Systemic	Acute	CIPN/Migraine/Neuropathic	Synergistic at sub-threshold doses (CIPN); 100:1 CBD:THC rescued migraine outcomes; sex-specific ratio effects (neuropathic)	King et al., 2017; Zorrilla et al., 2025; Borgonetti et al., 2023
THC + CBD (PK)	High CBD + Low THC (CBD 30 mg/kg + THC 0.3 mg/kg i.v.)	i.v.	Acute	Naïve	High-dose CBD ↑ THC brain/blood levels and potentiated antinociception; pharmacokinetic rather than pharmacodynamic mechanism	Varvel et al., 2006
THC + terpenes (full extract)	Moderate (4.2–14.5 mg/kg i.p.)	Systemic	Acute	Thermal/Visceral	Full extract = terpene-reduced for most endpoints; terpenes selectively enhanced supraspinal (hot-plate) antinociception	Harris et al., 2019
CBD-enriched extract (eCBD)	Moderate (10 mg/kg CBD-eq. oral × 7 days)	Oral	Chronic	Neuropathic	Outperformed matched pure CBD or THC; reversed thermal hyperalgesia; TRPV1-dependent; CYP450 inhibition may raise THC exposure	Comelli et al., 2008
Broad-spectrum cannabis oil	Low (0.1–3 mg/kg oral/i.pl./i.t.)	Oral/Local/Intrathecal	Acute/Chronic	Fibromyalgia-like	↓ mechanical hyperalgesia via all routes; chronic oral improved affective outcomes without locomotor impairment	Ferrarini et al., 2022
NEPE14 elixir	Moderate (6.6–20.7 mL/kg i.p.; 30–300 μL oral-mucosal)	Systemic/Oral-mucosal	Acute	Inflammatory	↓ thermal and mechanical hyperalgesia in CFA; no effect in naïve animals; no generalized sedation	Morris et al., 2024
THC + CBD (oral)	Low (THC 0.5–4 mg/15 mL + CBD 1 mg/15 mL gelatin)	Oral	Chronic	Neuropathic	↓ allodynia, hyperalgesia, and pain-related vocalizations over 2 weeks	Abraham et al., 2020

Dose is categorized as low, moderate, or high relative to each compound class: THC (low<1, moderate 1–10, high>10 mg/kg); CBD (low<10, moderate 10–40, high>40 mg/kg); Terpenes (low<10, moderate 10–100, high>100 mg/kg). Route is consolidated (systemic = i.p. or s.c.; local = intraplantar). CCI, chronic constriction injury; CFA, complete Freund’s adjuvant; CIPN, chemotherapy-induced peripheral neuropathy; i.t., intrathecal; i.v., intravenous; pSNL, partial sciatic nerve ligation; SNI, spared nerve injury; vlPAG, ventrolateral periaqueductal gray.

Beyond analgesia, individual components of THC:CBD combination products have therapeutic effects in other conditions, largely studied as monotherapies rather than blends. For example: antiemetic and appetite-stimulating effects of THC (Whiting et al., 2015), CB1/CB2 agonist-mediated benefit in atherosclerosis and cardiovascular disease (Pertwee, 2009), anticonvulsant effects of CBD (Devinsky et al., 2017), and neuroprotective effects in retinitis pigmentosa and other retinal neurodegenerative diseases (Rapino et al., 2018). Dedicated evidence for these effects using combined THC:CBD formulations specifically remains limited, and a systematic evaluation of these non-analgesic indications is beyond the scope of the present review.

### 2.2 Adult clinical pain

Clinical studies of cannabinoid use for pain relief show a wide range of efficacy depending on route of administration, dosing regimen, and pain type (Boehnke and Clauw, 2022; National Academies of Sciences, Engineering et al., 2017). *Cannabis*-based edibles are a common delivery mechanism for pain relief in patient populations, and oral CBD and THC have been shown to be efficacious for treating chronic pain on their own (Villanueva et al., 2022; Mohammed et al., 2024) as well as through a combined treatment (Hatav et al., 2025; Langford et al., 2013; Johnson et al., 2010). Here, we first summarize clinical data for oral, sublingual, and inhaled THC and CBD in chronic pain, then highlight emerging patterns in efficacy and tolerability. Chronic dosing of cannabis edibles and sublinguals reduce the symptoms of chronic pain of many etiologies, including back pain (Karst et al., 2025; Melendez et al., 2024), migraine (Cuttler et al., 2020), and more (Hatav et al., 2025). Studies vary in the use of different combinations of CBD/THC concentrations, as well as timing and duration of dosing, but generally show a reduction in pain symptoms in cannabis groups (see table). Taken together, these trials suggest that oral cannabinoids offer broad but heterogeneous benefit across chronic pain etiologies, with variability likely driven by underlying mechanisms and patient group. The variability in CBD/THC concentrations and timing/duration reflect the absence of standardized dosing guidelines, lack of purity regulations, and the challenge of balancing analgesia against psychoactive and cognitive side effects. Studies have shown that edible dosing produces relatively stable blood concentrations of THC for several hours after ingestion, with delayed onset and prolonged time to peak compared with cannabis inhalation (Johnson et al., 2025; Boehnke et al., 2024). This pharmacokinetic profile makes edibles particularly suited to chronic pain management, but also increases the risk of delayed and prolonged impairment if doses are not carefully titrated and regimented. Consistent with these findings, other oral formulations show similar patterns. For example, THC administration through sublingual or oral spray has also been shown to reduce pain symptoms in patient groups (Notcutt et al., 2004; Johnson et al., 2010; Langford et al., 2013; Überall, 2020; Agbaria et al., 2025).

Inhalation of THC as a method of acute dosing has been shown to improve chronic pain symptoms from a range of pain etiologies, with dosages ranging from low single-digit to mid-double-digit milligrams of THC (roughly corresponding to one to several inhalations of cannabis products) per session depending on product potency and inhalation protocol (see Table 2). Acute THC inhalation reduces symptoms of chronic neuropathic pain, and the rapid onset through inhalation may be advantageous for flares or breakthrough pain (Almog et al., 2020; Wallace et al., 2015; Wilsey et al., 2013; Andreae et al., 2015; Vulfsons et al., 2020). Doses are variable across studies and across groups, with higher potency cannabis generally producing more efficacious pain relief, at the cost of more frequent psychoactive and cognitive adverse events (Johnson et al., 2025; Almog et al., 2020; Wilsey et al., 2013). This pattern indicates that in inhalation-based regimens the therapeutic window is constrained by cognitive and psychomotor impairment.

**TABLE 2:** Clinical studies on the analgesic effects of cannabinoids.

Reference	Phytocannabinoid/Terpene	Dose	Route of Administration	Acute or Chronic	Pain Types	Effect
THC – Inhalation						
Almog et al., 2020	THC	0.5 or 1 mg (metered-dose inhaler)	Inhalation	Acute (single inhalation, crossover RCT)	Chronic neuropathic pain/CRPS (baseline pain ≥ 6 VAS)	Both doses significantly reduced pain intensity vs. baseline; rapid onset; no cognitive impairment at low doses
Wilsey et al., 2013	Cannabis (THC 1.29% and 3.53%)	1.29% or 3.53% THC; single session (Volcano vaporizer)	Inhalation (vaporized)	Acute (3-period crossover RCT, n = 39)	Neuropathic pain (mixed: SCI, CRPS, nerve injury, HIV neuropathy)	Both low and medium doses significantly reduced pain vs. placebo; no dose-dependent difference in analgesia; psychoactive and cognitive adverse effects dose-dependent
Wallace et al., 2015	Cannabis (THC 1%, 4%, and 7%)	Low (1%), medium (4%), or high (7%) THC; single session	Inhalation (vaporized)	Acute (4-period crossover RCT, *n* = 16)	Painful diabetic peripheral neuropathy	Dose-dependent reduction in spontaneous pain; 4% and 7% significant vs. placebo; 1% trend only; higher doses caused euphoria or somnolence; modest cognitive effects
THC – Sublingual						
Agbaria et al., 2025	THC (sublingual cannabis oil)	0.2 mg/kg body weight, single dose	Sublingual	Acute (single dose, crossover RCT, *n* = 23)	Fibromyalgia (chronic widespread pain)	THC significantly increased offset analgesia and decreased spontaneous pain vs. placebo
Notcutt et al., 2004	THC; CBD; THC:CBD (1:1)	Self-titrated; wide range of doses across patients	Sublingual spray	Chronic (12-week; open-label then randomized double-blind crossover, *n* = 34)	Chronic mainly neuropathic pain	THC and THC:CBD reduced VAS pain ratings vs. placebo; CBD alone did not reduce pain; sleep quality improved with all cannabinoid treatments
THC:CBD – Oromucosal Spray						
Johnson et al., 2010	THC:CBD (nabiximols); THC alone	∼22–32 mg/day THC + ∼20–30 mg/day CBD (8–12 sprays/day)	Oromucosal spray	Chronic (2-week parallel-group RCT; add-on to opioids; *n* = 177)	Advanced cancer pain (moderate–severe, inadequately controlled by opioids)	THC:CBD significantly reduced mean NRS vs. placebo and doubled ≥ 30% responders; THC alone not superior to placebo; opioid doses unchanged
Langford et al., 2013	THC:CBD (nabiximols)	2.7 mg THC + 2.5 mg CBD per spray; self-titrated	Oromucosal spray	Chronic (14-week parallel-group RCT; *n* = 339)	Central neuropathic pain in multiple sclerosis	Primary NRS endpoint not significant; pre-specified enrichment analysis: THC:CBD significantly delayed treatment failure vs. placebo (57% vs. 24% failed, *p* = 0.04); sleep improved
THC:CBD/Full-Spectrum Cannabis – Oral						
Haney et al., 2025	CBD + THC (100 mg CBD: 5 mg THC per capsule)	100 mg CBD: 5 mg THC TID (dose reductions allowed)	Oral	Chronic (8-week pilot RCT, *n* = 12)	Taxane-induced peripheral neuropathy (TIPN)	Cannabis group had significantly worse neuropathy ratings, sleep, and pain interference than placebo despite time-related improvement in both groups
Karst et al., 2025	Full-spectrum Cannabis sativa extract (VER-01)	2.5 mg THC per dose, titrated to max ∼20 mg THC/day	Oral	Chronic (12-week double-blind RCT + long-term extension, *n* = 820)	Chronic low back pain (CLBP), including neuropathic pain component	VER-01 significantly reduced mean NRS vs. placebo (-1.9 vs. -1.3); also improved neuropathic symptoms, physical function, and sleep
Cannabis (Varied Cannabinoid Profiles)						
Melendez et al., 2024	Cannabis (THC-dominant, CBD-dominant, or THC + CBD combined)	*Ad libitum*	Edible	Acute/Chronic (2-week prospective naturalistic study, *n* = 249)	Chronic low back pain	Pain intensity decreased over time across all product groups; THC-dominant products produced greatest acute pain reduction; significant correlation between THC dose and short-term pain relief
Cuttler et al., 2020	Cannabis (multiple product types tracked)	*Ad libitum*; tracked via Strainprint app	Multiple routes (primarily inhaled; also edibles and other)	Acute (real-world app-based naturalistic study)	Headache and migraine	∼47% reduction in headache severity; ∼49% reduction in migraine severity; concentrates more effective than flower; tolerance may develop with frequent use; men showed greater headache reduction than women
Hatav et al., 2025	Medical cannabis (varied THC/CBD + terpenoids)	Not standardized; individually prescribed products	Oral (usual patient use)	Chronic (8-week prospective observational, *n* = 329)	Chronic pain (mixed etiologies)	Cannabis chemical composition significantly predicted pain relief beyond demographics; specific terpenoids (α-bisabolol, eucalyptol) were key predictors of analgesia
Rapin et al., 2023	Cannabis-based medicines (varied THC/CBD profiles)	Individualized dosing; varied cannabinoid profiles	Oral (varied formulations)	Chronic (3-month prospective observational case series, *n* = 495)	Chronic non-cancer pain (mixed etiologies)	Significant improvements in pain severity, interference, and QoL at 3 months; THC-dominant recommended for severe/neuropathic pain; CBD-dominant for less severe; ADRs more common in cannabis-naive patients
CBD – Oral, Acute Dosing						
Arout et al., 2022	CBD	200, 400, or 800 mg (single dose)	Oral	Acute (crossover RCT, healthy adults, *n* = 17)	Experimental pain (cold pressor test)	CBD did not consistently improve pain threshold or tolerance vs. placebo; all doses actually increased ratings of painfulness (p<0.01)
Bebee et al., 2021	CBD	400 mg (single dose)	Oral	Acute (single dose RCT, emergency dept patients, *n* = 100)	Acute low back pain	CBD not superior to placebo at 2 or 12 h; no difference in adverse events between groups
De Vita et al., 2022	CBD	300 mg (single dose)	Oral	Acute (crossover, 2 × 2 balanced placebo design, *n* = 15)	Experimental pain (cold pressor and pressure pain)	CBD pharmacological effects and analgesic expectancy separately reduced pain unpleasantness; CPM improved with both; pain intensity (threshold/tolerance) not significantly changed by either
CBD – Oral, Chronic Dosing						
Capano et al., 2020	CBD (hemp extract)	∼30 mg/day for 8 weeks	Oral	Chronic (8-week prospective cohort, *n* = 97; no placebo)	Chronic pain (mixed etiologies) in patients using opioids	53% reduced or eliminated opioid use after adding CBD; improved pain scores and sleep quality; uncontrolled (no placebo comparison)
Pramhas et al., 2023	CBD	600 mg/day as add-on to paracetamol for 8 weeks	Oral	Chronic (8-week parallel-group RCT, *n* = 86)	Chronic knee osteoarthritis	CBD add-on not superior to placebo on WOMAC pain subscale or any secondary outcomes; liver enzyme elevations more frequent with CBD; well tolerated overall
CBD – Topical/Transdermal						
Heineman et al., 2022	CBD (topical)	∼6.2 mg/application (1 mL of 6.2 mg/mL), BID for 2 weeks	Topical	Chronic (2-week double-blind RCT, *n* = 18)	Thumb basal joint (CMC) arthritis	Topical CBD significantly reduced pain VAS and improved grip strength and DASH scores vs. placebo; well tolerated; no systemic adverse events
Walczyñska-Dragon et al., 2024	CBD (5% and 10% formulations)	5% or 10% CBD oromucosal/topical formulation for 30 days	Oromucosal/topical	Chronic (30-day 3-arm parallel-group RCT, *n* = 60)	Sleep bruxism–related muscular pain and jaw tension	Both 5% and 10% CBD significantly reduced pain intensity, muscular tension, and bruxism vs. placebo; 10% CBD produced greatest improvement; sleep quality improved
Bawa et al., 2024	CBD (transdermal gel)	4% w/w transdermal CBD gel, TID for 4 weeks	Transdermal	Chronic (4-week open-label feasibility trial, *n* = 15)	Hand osteoarthritis	Significant reduction in pain scores (AUSCAN, VAS) and improved hand function over 4 weeks; well tolerated; no serious adverse events; uncontrolled (no placebo)
Xu et al., 2020	CBD (topical oil)	250 mg CBD per 3 fl oz bottle applied for 4 weeks	Topical	Chronic (4-week placebo-controlled parallel-group RCT, *n* = 29)	Peripheral neuropathy (lower extremities)	Significant reduction in intense pain, sharp pain, cold and itchy sensations vs. placebo; improved sleep; no adverse events; symptomatic relief without systemic effects

Entries are grouped by compound class and route of administration, then ordered chronologically within groups. BID, twice daily; CBD, cannabidiol; CLBP, chronic low back pain; CMC, carpometacarpal; CPM, conditioned pain modulation; CRPS, complex regional pain syndrome; NRS, numeric rating scale; RCT, randomized controlled trial; SCI, spinal cord injury; THC, delta-9-tetrahydrocannabinol; TID, three times daily; TIPN, taxane-induced peripheral neuropathy; VAS, visual analog scale.

Clinical evidence for CBD-only products is more limited and shows a dosing- and time-course-dependent profile, particularly in chronic pain. Similar to preclinical studies, single-dose or acute dosing of CBD does not reliably produce pain relief in patient populations (Arout et al., 2022; Bebee et al., 2021; Cásedas et al., 2024; De Vita et al., 2022) and it has been reported that low-dose CBD alone does not produce consistent pain relief (Arnold et al., 2023 “The Safety and Efficacy of Low Oral Doses of Cannabidiol: An Evaluation of the Evidence – Arnold – 2023 – Clinical and Translational Science – Wiley Online Library,” n.d.). Trials in musculoskeletal and postoperative pain using acute oral or sublingual CBD have failed to show clinically meaningful analgesic effects of acute CBD dosing compared with placebo (Mohammed et al., 2024). In contrast, chronic dosing of moderate- to high-dose CBD has been shown to be more efficacious at relieving chronic pain in clinical populations (PubMed Central (PMC), n.d.; Capano et al., 2020), particularly in patients with musculoskeletal (Walczyñska-Dragon et al., 2024; Bawa et al., 2024; Heineman et al., 2022) or neuropathic pain (Xu et al., 2020). Long-term studies of chronic CBD dosing show meaningful pain reductions with weeks to months of CBD or CBD−dominant regimens (Rapin et al., 2023), although these studies are mixed (Pramhas et al., 2023) and also include frequent combination with THC. Acute CBD alone has not been shown to reliably relieve pain in humans, whereas chronic, higher-dose CBD shows more consistent analgesia in chronic pain conditions. These studies suggest that CBD’s potential analgesic effects may depend on chronic, high-dose exposure, potentially modulating inflammatory and neuromodulatory processes over time rather than providing rapid symptom relief.

An important consideration of *cannabis* administration for the treatment of pain is THC’s cognitive and physical impairment effects, as well as tolerance to THC during chronic use. Common adverse events include dizziness, cognitive impairment, fatigue, and psychoactive symptoms. These effects have been reported across chronic and acute clinical THC dosage regimens, including oral and inhaled preparations (Cuttler et al., 2020; Wilsey et al., 2013; Rapin et al., 2023; Bourque and Potvin, 2021; Notcutt et al., 2004). For instance, acute vaporized THC trials in neuropathic pain consistently document dose-dependent dizziness, feeling “high,” and slowed psychomotor performance, while chronic oral THC cohorts show persistent fatigue and complaints of memory loss (Johnson et al., 2025; Wilsey et al., 2013). Tolerance to THC’s analgesic and psychoactive effects has also been reported during long−term dosing, emphasizing the need for cautious titration and ongoing monitoring in chronic pain treatment (Cuttler et al., 2020; Rapin et al., 2023). Clinically, this can cause increased dose escalation over time, potentially increasing adverse effects while eroding the potential analgesic benefits. Cardiorespiratory outcomes across these studies are mixed: acute THC inhalation and oral CBD dosing in chronic pain patients have been associated with modest decreases in blood pressure and heart rate (Almog et al., 2020; Arout et al., 2022), but other analyses report increases in heart rate and blood pressure in some inhalation studies but not others (Andreae et al., 2015). Studies in healthy recreational users report increases in heart rate and blood pressure in THC-dominant but not CBD-dominant usage (Cheung et al., 2024), indicating that cardiorespiratory monitoring may be important in cannabinoid treatment for pain patients with cardiac conditions. In summary, while *cannabis* shows promise as treatment for chronic pain, its impairing effects and tolerance profile indicate a need for dosing strategies that balance efficacy against functional risks, particularly in chronic pain management where long-term use is required. In short, the phytocannabinoids THC and CBD show promising efficacy in treating several types of chronic pain in clinical settings, with small but clinically relevant improvements in pain and related outcomes for certain subsets of patients, which can be balanced against their adverse effects. The potential effects of terpene dosing in conjunction with phytocannabinoids or alone has been suggested (Hatav et al., 2025), but has yet to be studied in detail in clinical pain settings.

## 3 Novel methods in pain detection

Traditional rodent pain assays such as Von Frey, Hargreaves, and hot plate tests have been developed and utilized for decades as the gold standard for characterizing pain state and analgesia in rodent models of nociception. These assays yield withdrawal thresholds across sessions, under controlled conditions, and they are sensitive to dose-dependent effects of clinically used analgesics (Turner et al., 2019; Modi et al., 2023; Deuis et al., 2017). However, these methods are also sometimes inconsistent across labs, low-dimensional and often experimenter or approach dependent limiting their translational value. This makes it difficult to disentangle analgesic mechanisms from other behavioral profiles like motor or cognitive impairment. By typically reducing pain to a single binary or latency-based endpoint (withdrawal vs. no withdrawal, or time to withdrawal), we cannot capture the richness of pain-related behaviors or drug efficacy. Reviews of rodent pain assessment have emphasized that these approaches oversimplify pain into a single reflex output and cannot capture spontaneous pain (Turner et al., 2019; Tappe-Theodor et al., 2019; Deuis et al., 2017). As a result, impairment and affective dimensions of pain are under−sampled, making it challenging to balance analgesic benefit against motor and cognitive side effects, particularly for cannabinoids. Machine-learning–based pose-estimation tools such as DeepLabCut and SLEAP allow high-resolution tracking of multiple body parts in freely moving rodents, enabling objective quantification of complex and subtle behavioral states over long timescales. The recent advent of machine learning–based behavioral analysis pipelines have begun to provide a rich, unbiased, and objective framework that is well suited to understanding the therapeutic and impairing effects of cannabinoids in preclinical pain models. Supervised and unsupervised pipelines (SimBA, BSOiD, VAME, MoSeq, Keypoint-MoSeq) go beyond manual scoring to discover naturalistic, spontaneous, and evoked behaviors, and transitions between states and “behavioral syllables” (Luxem et al., 2023; Bumgarner et al., 2022; Wiltschko et al., 2020; Weinreb et al., 2024). These approaches can quantify how behavior state structure, frequency, and transitions change with pharmacological manipulation (Wiltschko et al., 2020). These methods are especially useful for analgesic studies because they can reveal drug-specific effects on spontaneous behavior, locomotion, and postural dynamics that are not captured by withdrawal-based assays alone.

For analysis of pain behaviors, machine-learning tools are beginning to transform analysis through extraction of kinematic features that reflect both evoked and ongoing pain states. They can be used to build automated pain scales by quantifying paw withdrawal height, trajectory, and guarding duration, as well as naturalistic pain behaviors such as licking, biting, and grooming (Bumgarner et al., 2022; Chen et al., 2025 “Deep Learning-Based Animal Behavior Analysis: Insights from Mouse Chronic Pain Models,” n.d.). Measures of facial expressions also provide a more objective readout of spontaneous and affective pain in rodents. Facial action units (FAUs) are movements of certain facial muscles, and are associated with the presence and intensity of pain in rodents. Validated grimace scales like the Mouse Grimace Scale and the Rat Grimace Scale score FAUs to infer pain state. These methods have relied on manual scoring of facial movements and expressions, but have also recently been shifting toward automated quantification of facial expressions using neural networks (Domínguez-Oliva et al., 2022; Tuttle et al., 2018; Sotocinal et al., 2011), and combining these data with classification of pain-evoked body postures (Seicol et al., 2026). Other work has shown that unsupervised clustering of pose-derived features can reveal pain-linked changes in rearing, climbing, posture, and gait, and transitions between behavioral states that are not captured by traditional scoring (Bumgarner et al., 2022; Jones et al., 2020). For instance, pose-estimation can differentiate a fast, reflexive flick from a sustained guarding posture or limping gait, even when both would be scored as a ‘withdrawal’ in traditional assays. Further, these pipelines track behavior continuously over minutes to hours, rather than only at the instant of stimulus delivery, allowing spontaneous and ongoing pain states to be quantified alongside evoked responses.

**FIGURE 2:**
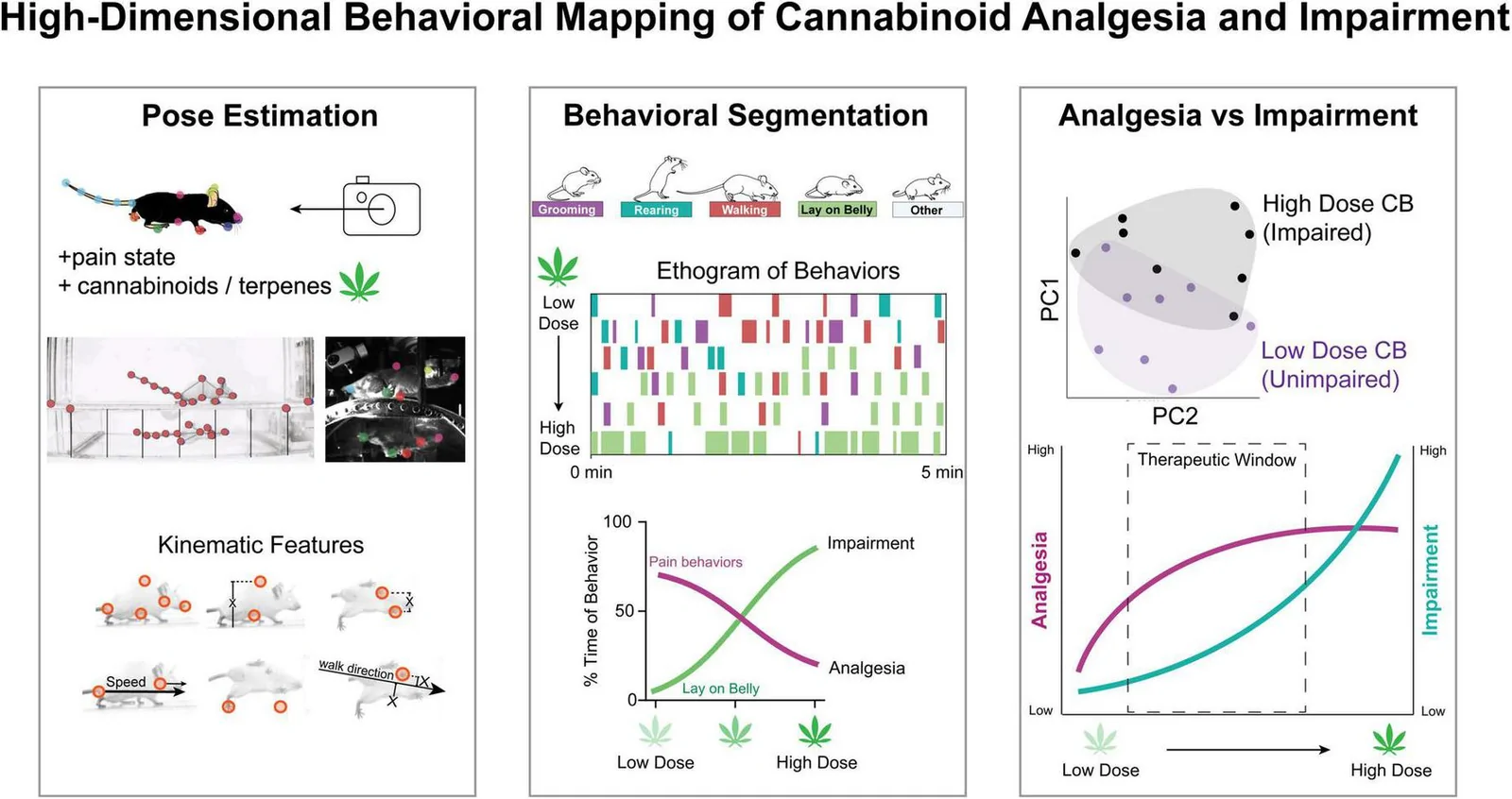
High-dimensional behavioral mapping of cannabinoid analgesia and impairment. Machine learning approaches for behavioral analysis measure cannabinoid analgesia versus impairment. Pose estimation (left): tracking of body points via machine learning extracts kinematic features including speed, body angle, and walk direction from freely moving animals administered cannabinoids in a pain state. The approach generates continuous, high-resolution behavioral data without observer bias. Behavioral segmentation (middle): kinematic features are used to classify behavior into discrete ethogram categories (grooming, rearing, walking, lay on belly, and other), visualized across time for low- and high-dose cannabinoid conditions. As dose increases, time spent in pain-associated behaviors (e.g., lay on belly) decreases while impairment-associated behaviors increase, tracing separable dose-response curves. Analgesia vs. impairment (right): dimensionality reduction via principal component analysis separates low-dose (unimpaired) from high-dose (impaired) cannabinoid conditions in behavioral space, and plotting analgesia and impairment as independent dose-response functions reveals a therapeutic window: a dose range in which analgesia is achieved before impairment emerges. These approaches provide a quantitative framework for evaluating the therapeutic potential of cannabinoids and terpenes in preclinical pain models (Kinematic features and behavioral poses adapted from English et al., 2025).

Machine−learning behavioral pipelines represent an innovative approach to study how cannabinoids differentially alter pain, motor function, and emotional state across doses and development of pain responses. In this context, these methods make it possible to quantify analgesia while also tracking impairment metrics such as gait disruption, ataxia, or hypoactivity, providing a way to separate benefit from adverse effects and to compare behavior directly with neural readouts (English et al., 2025; Jones et al., 2020). By combining supervised pose−estimation and unsupervised classification of behaviors, one can map dose-response relationships for cannabinoid−induced analgesia while also quantifying “impairment” (like sedation, ataxia, reduced exploration) in juveniles, adolescents, and adults (English et al., 2025). This enables the identification of therapeutic windows wherein analgesic benefit is maximized and adverse effects are minimized (Figure 2). This approach allows the separation of beneficial analgesic effects of cannabinoids from the motor and neurodevelopmental consequences, improving the translational relevance of preclinical cannabinoid pain research. In addition, digital twin technology (Attaran and Celik, 2023; Kumar et al., 2022), in which behavioral and biological readouts can be integrated into a virtual model that mirrors an animal’s real-time pain state and drug response, may be a useful next step in cannabinoid and pain research. In preclinical cannabinoid studies, digital twin frameworks could combine analgesic behavioral data, impairment metrics, and neural data to simulate how an animal might respond across dose, age, or developmental stage, enabling *in silico* exploration of therapeutic windows versus adverse effects. In this sense, digital twins complement machine-learning behavioral pipelines by advancing to a predictive, iterative model of analgesia versus impairment that may improve translational relevance. Overall, machine-learning behavioral pipelines represent the next progressive step in understanding pain, analgesia, and the effects of cannabinoids in preclinical studies.

Zhang et al., 2022 combined whole-body pose tracking with machine vision analysis of paw-surface contact to extract dynamic behavioral features and generate a paw luminance ratio measure that is sensitive to mechanical hypersensitivity across time. This approach showed that pain-related behavioral changes were reversible with analgesics and could be objectively differentiated from sedation, making this approach highly relevant for preclinical cannabinoid studies where separating true antinociception from motor impairment is essential (Zhang et al., 2022). Oswell et al., 2026 developed Light Automated Pain Evaluator (LUPE), a deep learning behavioral platform integrating DeepLabCut pose estimation with supervised and unsupervised machine-learning algorithms (A-SOiD and B-SoiD) to classify spontaneous pain-related behaviors in freely moving mice. Using LUPE, they identified distinct behavioral states associated with acute and chronic pain and demonstrated that morphine selectively reversed affective pain behaviors without altering reflexive sensory responses. Further, they used these behavioral and neural signatures to develop an opioid-based chemogenetic strategy that mimicked morphine-like analgesia through targeted inhibition of opioid-sensitive anterior cingulate cortex ensembles (Oswell et al., 2026). Together, these approaches provide a powerful translational framework for distinguishing true analgesia from non-specific behavioral suppression and are relevant for studying cannabinoid-induced analgesic and impairment profiles in preclinical models. English et al., 2025 developed a video-monitored behavioral platform with machine learning classifiers to characterize discrete changes in natural mouse behaviors and the effects of phytocannabinoid dosing. They used this experimental approach to demonstrate that machine-learning behavioral analysis can be leveraged to characterize THC-induced changes in naturalistic behaviors, including impairment of walking. Further, they used a computational approach wherein THC-specific dose prediction algorithms decode the “presented behavioral dose” of manipulated mice (English et al., 2025). Combined, these studies uncover novel cannabimimetic behavioral signatures, and provide a new lens to study behavioral profiles of impairment in rodents that can be extrapolated to pain and other drug fields.

In human clinical studies, segmentation of discrete behavioral movements is used to characterize gait and posture in certain types of pain state and musculoskeletal disorder (Mizuguchi et al., 2024; Moggioli et al., 2025; Dion et al., 2024). Using these techniques, and drawing from preclinical studies using machine-learning based behavioral analysis, clinical cannabinoid trials have begun to move beyond coarse scales (pain ratings, “sedated/not sedated”) toward kinematic readouts of function and side effects. Machine learning in human kinematics is being used to define impairment as a result of cannabis dosing. For example, driving safety studies integrate driver-monitoring systems to extract ocular and movement-related markers of cannabis impairment, with machine-learning used to identify patterns indicative of being too impaired to drive (Driving Safety Research Institute, 2022 “Detecting Impairment from Cannabis | Driving Safety Research Institute – College of Engineering | The University of Iowa,” n.d.). Deep-learning “digital gait” work like MariaGait, uses smartphone accelerometer/gyroscope gait time-series transformed into images and a neural network to discriminate alcohol-induced versus marijuana-induced gait impairment (Li et al., 2025 “Discriminating Between Marijuana and Alcohol Gait Impairments Using Tile CNN With TICA Pooling” 2025). Clinical cannabinoid research for pain treatment will be progressed by systematically using similar machine-learning pipelines in cannabinoid clinical trials to map analgesic benefit and impairment in the shared, high-dimensional human kinematic space.

## 4 Discussion

Cannabinoid-based analgesia is constrained by many variables including chemical composition, dose, route of administration, treatment chronicity, pain etiology, biological confounds (i.e., sex, prior exposure), and drug-drug interactions (i.e., “entourage effect”). All of these variables working together explain why cannabis-pain research has historically produced heterogenous results. A few results are consistent, however: (1) THC is a reliable analgesic across pain types, but its therapeutic window is narrow because of the dose overlap of analgesia and psychomotor-impairment. Intrathecal delivery partially dissociates spinal anti-hyperalgesia from the CB_1_R mediated tetrad. (2) CBD’s pharmacological profile is quite different where its acute effects are limited and inconsistent, but repeated administration in neuropathic and chemotherapy-induced pain is efficacious. This has direct implications for future clinical trial design. (3) Terpenes like BCP, linalool, and myrcene, contribute independent antinociceptive effects, and machine-learning analyses of cannabis use suggest that terpenoids composition more than THC or CBD content alone can predict analgesic response.

Of all of the variables, two stand out as the most tractable levers for improving preclinical-to-clinical translation. The first is route of administration and formulation. Delivery strategies that favor ideal pharmacokinetics (i.e., extended-release oral drugs) can reduce the peak-concentration side effects that limit THC’s therapeutic index in most clinical formats. At the receptor level, the rational design of Gi-biased and peripherally restricted CB1 agonists offers a complementary pharmacological route to the same goal. Recent structure-guided compounds maintain analgesic efficacy across inflammatory, neuropathic, and migraine pain models while reducing hypolocomotion, hypothermia, tolerance, and rewarding properties in preclinical models (Liao et al., 2026; Rangari et al., 2025). This suggests that widening the CB1 therapeutic window may not require formulation solutions alone. The second lever is machine-learning behavioral phenotyping. Classical pain assays are the gold standard for studying evoked pain responses in rodents, but in order to reliably discern analgesia from sedation or motor impairment for cannabinoids whose CB_1_R effects confound these readouts, these traditional assays must be combined with novel pain detection methods to better understand naturalistic and spontaneous pain behaviors. Pose-estimation and unsupervised clustering pipelines can quantify kinematic signatures of guarding and gait asymmetry as well as define novel pain specific phenotypes that cannot be picked up by human eyes alone. We are now poised to map these features alongside evoked responses and map analgesia and impairment on common axes. As high-dimensional kinematic approaches start to be used in clinical cannabinoid trials, we will – for the first time – place preclinical and clinical dose-response results in a shared metric dimensional space which will directly address the translational gap that has limited progress in this domain to date.

This review has several limitations. First, by design we chose to focus exclusively on THC, CBD, and Cannabis-derived terpenes to have an in-depth exploration of dose, route, and chronicity. Minor Phytocannabinoids (e.g., CBG, CBN, CBC), CBDA, and synthetic cannabinoids (e.g., dronabinol, nabilone, rimonabant) were excluded and warrant a separate, dedicated review. Second, the preclinical work discussed is heterogeneous regarding specifies, sex, pain model, and dosing regimen, which limits cross-study comparison. Third, nearly all the mechanistic data for terpene and entourage effects was done in preclinical work and robust mechanistic confirmation in humans remains to be seen. Lastly, clinical trial data for cannabinoid analgesia continues to be affected by variable product standardization, blinding challenges, and inconsistent outcome measures across studies. All of this further complicate translation from preclinical findings.

Closing the gap from bench to bedside will depend on two main efforts: (1) formulation and pharmacological strategies that widen the therapeutic window between analgesia and CB1R-mediate side effects (e.g., extended-release designs or Gi-biased CB1 agonists), and (2) adoption of high-dimensional, machine-learning-based behavioral phenotyping to precisely dissociate analgesia from sedation and motoro impairment. Standardizing dose, route, and composition reporting across studies will be imperative to build more complete picture of cannabinoid analgesic efficacy. Taken together, the studies reviewed here demonstrate that the analgesic efficacy of THC, CBD, and Cannabis-derived terepenes is dependent on dose, route of administration, treatment chronicity, and chemical composition. THC typically offers reliable but a narrow-window analgesia, while CBD shows efficacy with repeated dosing in chronic pain states, and terpenes contribute independent and composition-dependent effects. Addressing the translational gap in this field will require refined pharmacological strategies and more sensitive behavior tools.
